# Determining the role of missense mutations in the POU domain of HNF1A that reduce the DNA-binding affinity: A computational approach

**DOI:** 10.1371/journal.pone.0174953

**Published:** 2017-04-14

**Authors:** Sneha P., Thirumal Kumar D., George Priya Doss C., Siva R., Hatem Zayed

**Affiliations:** 1 School of BioSciences and Technology,Vellore Institute of Technology, Vellore, Tamil Nadu, India; 2 Department of Biomedical Sciences, College of Health Sciences, Qatar University, Doha, Qatar; Bioinformatics Institute, SINGAPORE

## Abstract

Maturity-onset diabetes of the young type 3 (MODY3) is a non-ketotic form of diabetes associated with poor insulin secretion. Over the past years, several studies have reported the association of missense mutations in the Hepatocyte Nuclear Factor 1 Alpha (HNF1A) with MODY3. Missense mutations in the POU homeodomain (POU_H_) of HNF1A hinder binding to the DNA, thereby leading to a dysfunctional protein. Missense mutations of the HNF1A were retrieved from public databases and subjected to a three-step computational mutational analysis to identify the underlying mechanism. First, the pathogenicity and stability of the mutations were analyzed to determine whether they alter protein structure and function. Second, the sequence conservation and DNA-binding sites of the mutant positions were assessed; as HNF1A protein is a transcription factor. Finally, the biochemical properties of the biological system were validated using molecular dynamic simulations in Gromacs 4.6.3 package. Two arginine residues (131 and 203) in the HNF1A protein are highly conserved residues and contribute to the function of the protein. Furthermore, the R131W, R131Q, and R203C mutations were predicted to be highly deleterious by *in silico* tools and showed lower binding affinity with DNA when compared to the native protein using the molecular docking analysis. Triplicate runs of molecular dynamic (MD) simulations (50ns) revealed smaller changes in patterns of deviation, fluctuation, and compactness, in complexes containing the R131Q and R131W mutations, compared to complexes containing the R203C mutant complex. We observed reduction in the number of intermolecular hydrogen bonds, compactness, and electrostatic potential, as well as the loss of salt bridges, in the R203C mutant complex. Substitution of arginine with cysteine at position 203 decreases the affinity of the protein for DNA, thereby destabilizing the protein. Based on our current findings, the MD approach is an important tool for elucidating the impact and affinity of mutations in DNA-protein interactions and understanding their function.

## Introduction

Maturity-onset diabetes of the young (MODY) is a hereditary monogenic form of diabetes, with eleven different forms caused by changes in eleven different genes, of the eleven forms, MODY2 and MODY3 are the most common; with frequent mutations in the *GCK* and *HNF1A* genes [1–4]. The diagnosis of MODY3 is clearer during adolescence or early adulthood and also requires pharmacological treatment. Patients with MODY3 are known to develop late-onset microvascular complications [5, 6]. Furthermore, MODY3 is defined as a non-ketotic and autosomal dominantly inherited form of diabetes characterized by a severe deficiency in insulin secretion. Heterozygous mutations in the *HNF1A* gene are further transcribed to produce the protein (transcription factor), leading to a confirmed disease condition [7].

**Fig 1:**
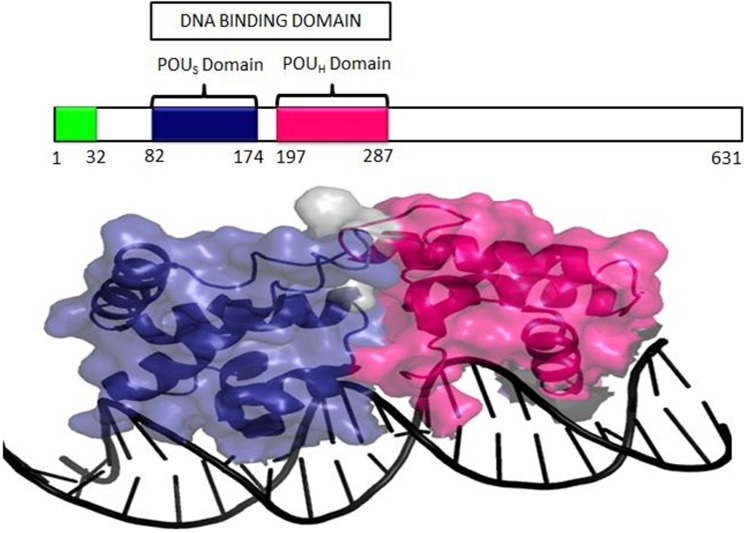
Three-dimensional structure of the HNF1A protein (PDB ID-1IC8) shows the POU_H_ (marked in pink) and POU_S_ (marked in blue) domains interacting with DNA. The protein is depicted as a cartoon-surface and DNA in black cartoon strand using PyMOL software.

Hepatocyte Nuclear Factor 1 Alpha (HNF1A), also known as TCF1 (Hepatic Transcription Factor 1), belongs to the POU transcription factor family, which is highly expressed in the liver, pancreatic β-cells and kidney [8–10]. The *HNF1A*gene is located on chromosome (12q24.2), spanning 23,790 bp, and it encodes a 631 amino acid-long protein consisting of an amino-terminal dimerization domain (residues 1–32), a DNA-binding motif containing a typical homeodomain (residues 203–276), and a carboxyl-terminal transactivation domain (residues 281–631) [11]. Chi et al. [2002] co-crystallized the human HNF1A protein (83–279 amino acids) and showed that the protein binds to the promoter of target genes as a dimer [12]. The protein consists of two domains, namely, a homeodomain and another domain that is structurally similar to the POU domain. The highly conserved POU domain is further divided into two sub domains, POUs (specific domain) and POU_H_ (homo domain) (Fig 1) [13, 14]. POUs is an integral part of HNF1A that helps in maintaining the stability of the protein, whereas the POU_H_ domain of the transcription factor acts as a crucial interface initiating the interaction between the protein and DNA[12,15].

Generally, DNA-protein interactions have pivotal roles in important cellular mechanisms, such as transcription and gene regulation [16]. DNA-binding proteins have specific DNA-binding domains. Therefore, they have a higher affinity for DNA and play a significant role in gene regulation. Genetic mutations in the DNA-binding homeodomain of the protein have recently attracted much attention, as they are related to various human diseases associated with developmental and metabolic disorders [15, 17–19], which affects transcription factor function, leading to abnormal control of the transcriptional machinery [12] and thereby directly hindering the function of the protein [20]. In particular, mutations in the HNF1A expressed in pancreatic cells lead to β-cell dysfunction, causing diabetes mellitus (MODY3); moreover, individuals carrying HNF1A mutations represent approximately 2% of overall diabetes cases [21,22]. According to the Human Gene Mutation Database (HGMD) [23] (http://www.hgmd.org/), the majority of the mutations detected in individuals with MODY3 were missense, 39 missense and five nonsense mutations that are located in the DNA-binding domain (POU_H_). Most of the mutations were observed in exons 1, 4, 6 and 10.

Several assays have been developed to understand the binding of the transcriptional factors (proteins) to DNA, such as the ChIP (chromatin immuno precipitation) assay [24], EMSA (electrophoretic mobility shift assay) [25], DNA pull-down assay [26], DNAse footprinting assay [27], and mass spectrometry [28]. Although these well-developed assays can identify the residues in proteins that interact with the DNA, computational methods have significantly evolved to decrease the burden of experimental analysis in terms of cost and time and to improve the accuracy. In the present study, we utilized online databases to retrieve the reported mutations in HNF1A. Sequence and structure-based methods or combined methods with diverse algorithms, namely, PhD-SNP [29], fathmm [30] Align GV-GD [31], SNAP [32], PON-P2 [33], PolyPhen-2 [34], HANSA [35], SIFT [36], PANTHER [37], and SNPs&GO [38], were applied to determine the pathogenic impact of the missense mutations, i.e., impact on protein functions. Protein stability and function are interdependent, and an aberration in either one of the phenomena directly affects the other. Therefore, mutations that are destabilizing the protein were identified using I-Mutant 2.0 [39], MUpro [40], and DUET [41]. The primary focus of the study is to understand the binding patterns of the mutant proteins to DNA. In this context, the DNA-binding sites of the protein were detected using the BindN+ tool [42], and the observed data were cross-validated using PDBsum. Furthermore, CoCoMaps [43] was used to identify the interaction sites between the two macromolecules (protein and DNA), and the difference in the number of salt bridges formation in the protein molecules (native and mutant) was calculated using the ESBRI web server tool [44].A molecular docking analysis was performed using the HADDOCK server to analyze the binding efficiency of the native and mutant proteins toward DNA [45]. Finally, the docked complexes of the mutations (R131W, R131Q and R203C) that showed an impact on the protein function and decreased the DNA-binding efficiency were subjected to molecular dynamics (MD) simulations using the GROMACS v4.6.3 package [46]. This simulation analysis was performed in triplicate, allowed us to explain the impact of the mutation at the molecular level and also verified the results obtained from the *in silico* prediction methods. The computational workflow described here for studying the structural and functional impacts of HNF1A missense mutations on DNA-protein interaction sites can be easily implemented in a pipeline for any other DNA-protein interactions (S1A and S1B Fig).

## Results

### Retrieval of mutations and pathogenic analysis

The effects of mutations associated with the HNF1A protein are of substantial clinical importance because they are known to be associated with MODY3 [47]. Therefore, all HNF1A missense mutations were retrieved from the dbSNP, UniProt, and HGMD databases. 219 missense mutations were analyzed with various *in silico* prediction tools to measure their effects on pathogenicity and stability. Among the *in silico* prediction tools, fathmm predicted that all the 219 missense mutations (100%) as deleterious (S1 Table), followed by HANSA (95.68%), SNAP (81.03%), PONP2 (51.72%), PolyPhen 2 (71.55%), PANTHER (58.62%), PhD-SNP (68.96%), and Align GV-GD (57.75%) (S2 Fig). However, SNPs&Go and SIFT predicted 35.34% and 12% of the missense mutations as deleterious.

### Screening of destabilizing mutations

Protein stability is a crucial aspect for maintaining proper function. The missense mutations that could destabilize the protein were analyzed using the algorithms; I-Mutant 2.0, MUpro, and DUET to increase the accuracy of the predictions. Eight missense mutations (N127Y, R131Q, R131W, S142F, R159W, R200W, R203C, and R263C) predicted to be the most deleterious (Table 1) by all the *in silico* prediction tools were subjected to the stability analysis. I-Mutant 2.0 predicted that the eight missense mutations had a destabilizing effect on the protein. MUpro predicted that N127Y, S142F, and R200W increased the stability, whereas the other five missense mutations, R131Q, R131W, R159W, R203C and R263C decreased the stability of the protein. The DUET, which incorporates two other analysis methods (mCSM & SDM), predicted four missense mutations, R131Q, R131W, R159W, and R203C, having destabilizing effects on the protein (Table 2).

**Table 1:** List of potential pathogenic HNF1A missense mutations predicted by all *in silico* prediction tools.

Mutation	PhD-SNP	Align GVGD	SNAP	PON-P2	PolyPhen-2	HANSA	SIFT	PANTHER	SNPs&GO	fathmm
N127Y	D	C65	NN	P	PrD	D	D	D	D	-5.72
R131Q	D	C65	NN	P	PrD	D	D	D	D	-5.77
R131W	D	C65	NN	P	PrD	D	D	D	D	-5.91
S142F	D	C65	NN	P	PrD	D	D	D	D	-5.74
R159W	D	C65	NN	P	PrD	D	D	D	D	-5.91
R200W	D	C65	NN	P	PrD	D	D	D	D	-4.35
R203C	D	C65	NN	P	PrD	D	D	D	D	-5.53
R263C	D	C65	NN	P	PrD	D	D	D	D	-4.99

PhD-SNP, HANSA, SIFT, PANTHER & SNPs&Go: D-deleterious; Align GVGD: C-class; SNAP: NN-non-neutral; PolyPhen-2: PrD-probably damaging; PON-P2: P-pathogenic; fathmm scores below 0 are deleterious.

**Table 2:** Prediction of the effects of HNF1A missense mutations on protein stability.

Mutation	Mupro (SVM)	I-Mutant 2.0 (kcal/mol)	DUET (kcal/mol)
N127Y	0.77534	-0.04	0.01
R131Q	-1	-1.15	-0.732
R131W	-0.344	-0.42	-0.991
S142F	0.741	-0.55	0.307
R159W	-0.061	-0.54	-0.319
R200W	0.334	-0.77	0.02
R203C	-0.4437	-0.35	-0.402
R263C	-0.304	-1.55	2.042

The numerical value denotes the 'delta delta G’ (DDG). A DDG value<0 indicates a decrease in stability, and DDG value >0 indicates an increase in stability.

### Residues of the protein involved in binding DNA

Binding of transcription factors to DNA regulates or deregulates a gene in a highly specific manner. The prediction programs PDBsum and BindN+ predicted 15 and 40 DNA-binding sites in HNF1A, respectively (S2 Table). Thirteen positions in HNF1A (POUs- R131Q, R131W, S142F, H143Y, K155R, and K158N; POU_H_- R203C, R203H, K205Q, R263C, R272C, R272H, and K273E) were described as DNA-binding sites by both programs. As a next step, all 13 DNAbinding sites were cross checked according to their potential deleteriousness, i.e., pathogenicity, and destabilizing effects reported by the various *in silico* prediction methods (Table 1 and Table 2). Based on these observations, we conclude that only three missense mutations R131Q, R131W, and R203C lie in the DNA-binding region, with highly deleterious and destabilizing effects, and thus, these mutations were selected for further structural analyses. R131Q and R131W reside in the POUs domain, whereas R203C resides in the POU_H_ domain.

### Sequence conservation analysis

**Fig 2:**
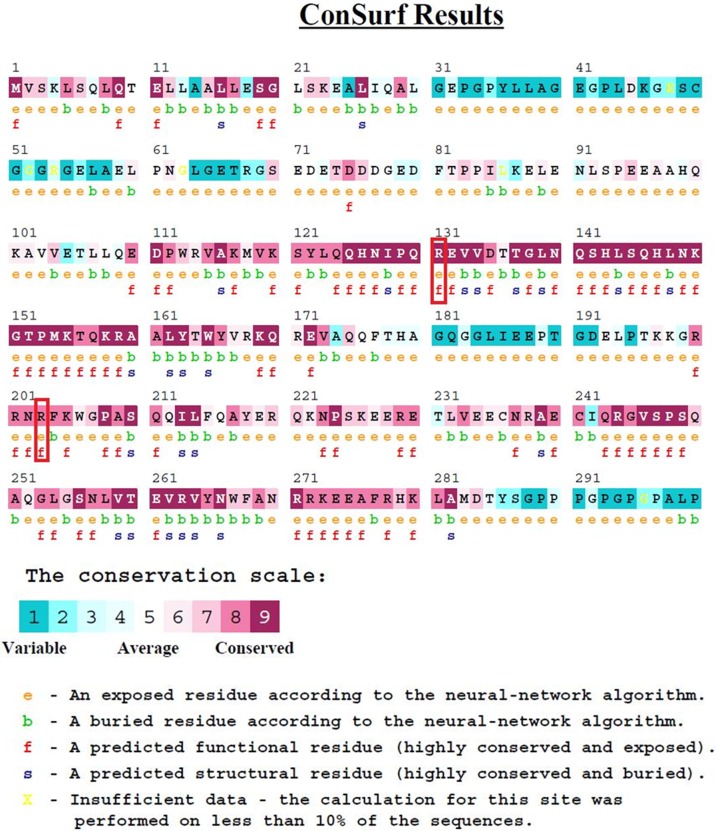
The ConSurf analysis. The arginine residues at positions 131 and 203 were highly conserved, with scores of 9 and 8, respectively.

The conservation analysis was performed with ConSurf to predict the conservation frequency of the R131W, R131Q, and R203C mutations in HNF1A. The amino acid arginine at both positions 131 and 203 was predicted to be a highly conserved region with scores of 9 and 8, respectively; these amino acids are also exposed (Fig 2), indicating that both amino acid positions had an impact on protein function. Fig 2 shows the positions of R131 and R203, which are located in the exposed region and interact with DNA. Based on the results obtained from ConSurf, we conclude that the mutations at the R131 and R203 positions could affect the protein function.

### Molecular docking analysis

The native and mutant proteins were subjected to a molecular docking analysis with DNA using the HADDOCK server. Based on the scores, the HADDOCK server yielded 10 different clusters of native and mutant complexes as the most reliable complexes with better binding efficiencies. Of the observed clusters, cluster number one of the native complex showed higher contributions of van der Waals and electrostatic energies of -154.1 kcal/mol and -657 kcal/mol, respectively. Meanwhile, mutant complexes R131W, R131Q and R203C exhibited van der Waals energies of -140.6, -142.1, and -144.33 kcal/mol, respectively. The observed electrostatic energy values were similar for the R131W and R131Q mutant complexes, with a value of -654 kcal/mol, but the R203C mutant complex showed a smaller electrostatic energy of -649 kcal/mol (Table 3).

**Table 3:** Docking analysis scores for native and mutant (R131W, R131Q, and R203C) complexes from the HADDOCK server.

Protein complexes	Binding Score	Van der Waals energy (kcal/mol)	Electrostatic energy (kcal/mol)	Desolvation Energy(kcal/mol)
Native	-279.34	-154.1	-657	3.4
R131Q	-276.45	-140.6	-654	-1.22
R131W	-277.56	-142.1	-654	-1.32
R203C	-273.33	-144.33	-649	-1.38

### Analysis of hydrophobic and hydrophilic interactions

The contacts at the interface between the amino acids present in the protein and DNA macromolecules were further elucidated using CoCoMaps. Variations in the number of hydrophilic-hydrophilic interactions were observed between the native and three mutant complexes. The native complex exhibited 181 hydrophilic-hydrophilic interactions. The R131W mutant complex participated in 183 interactions with fewer deviations. Meanwhile, the other two mutant complexes, R131Q and R203C, showed significant variations in the number of hydrophilic-hydrophilic interactions of approximately 201 and 202, respectively, clearly indicating a reduction in the hydrophobicity of the mutant complexes (S3 Table).

### Salt bridge analysis

The number of salt bridges formation between the native and the mutant complexes were calculated using the ESBRI online server by providing the atomic coordinates of each complex as the input. The basic phenomenon of salt bridge formation depends on the ionization properties of the amino acids and is also significantly influenced by the environment of the protein. Thirteen salt bridges were formed in the native complex. When comparing the numbers of salt bridges formed in the mutant complexes (R131W, R131Q, and R203C), we observed a similar number of salt bridges formation in R131W and R131Q mutant complexes and the native complex. However, in the R203C mutant complex, only 5 salt bridges formation was observed (S4 Table).

### Molecular dynamics (MD) simulations

**Fig 3:**
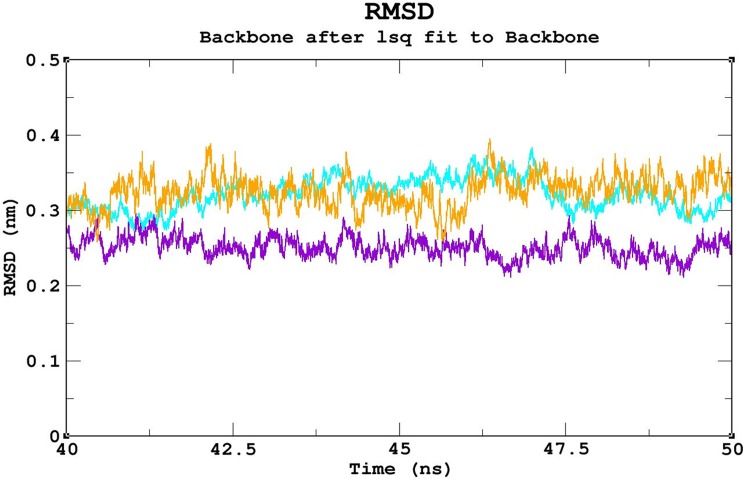
Block-based RMSD analysis of the native protein-DNA complex. Color scheme: run 1 (orange), run 2 (violet), and run 3 (cyan). For triplicate runs, trajectory files between 40ns and 50ns were used to elucidate the pattern with the greatest convergence. Trajectory run 2 (violet) shows the best convergence pattern.

**Fig 4:**
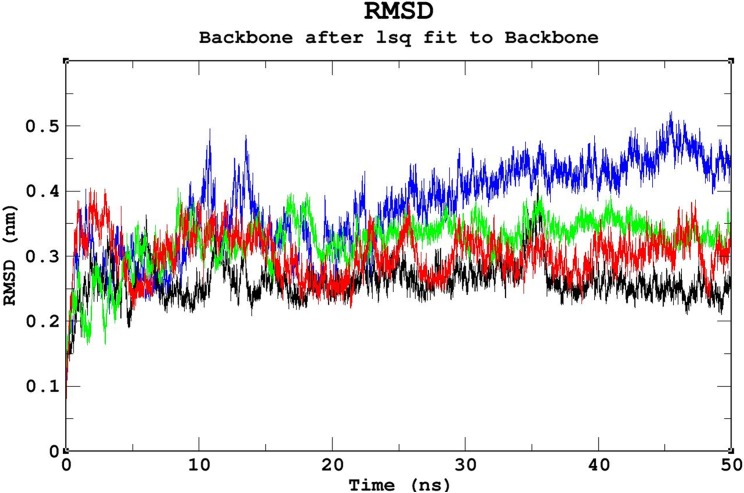
Backbone RMSD graph of the native and mutant complexes. Color scheme: native (black), R131W (red), R131Q (green), and R203C (blue). The native complex showed the pattern with the least deviation, the R203C mutant complex showed the pattern with the greatest deviation, and the R131W and R131Q mutant complexes showed intermediate deviation patterns between the patterns of the native and R203C mutant complexes.

**Fig 5:**
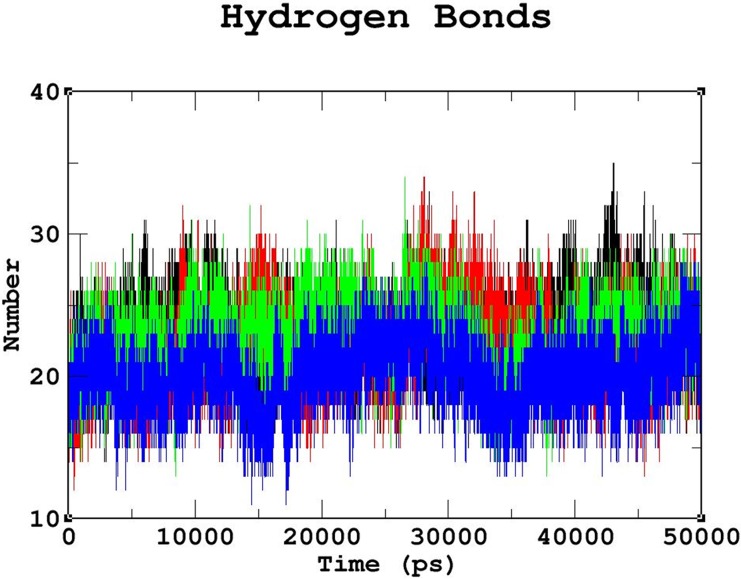
Number of intermolecular hydrogen bonds formed between the protein and DNA in the native and mutant complexes. Color scheme: native (black), R131W (red), R131Q (green), and R203C (blue).The R203C mutant complex formed the least number of intermolecular hydrogen bonds throughout the simulation period compared to the native complex, followed by the R131Q mutant complex. The R131W mutant complex formed less number of intermolecular hydrogen bonds than the native complex but more than the R131Q and R203C mutant complexes.

Triplicate runs of 50ns MD simulations using Gromacs were performed to understand the stability, functionality, and folding variations between the native and mutant complexes at an atomic level. Trajectory files obtained from the triplicate runs of the native complex were utilized to construct a Block-based root-mean-square deviation (RMSD) plot. This analysis allows us to compare and identify the most highly converged trajectory of the native complex for further analysis (Fig 3). Among the three runs, the native complex marked in violet (run 2) showed better convergence pattern (Fig 3). Runs 1 & 3 exhibited greater deviation pattern and lower convergence in the Block-based RMSD plots, which were used to plot the RMSD graph illustrated in S3A and S3B Fig. Trajectory files from run 2 of the native and mutant complexes were utilized to measure the impact of the mutations on protein-DNA interactions using various Gromacs utilities. The RMSD analysis was used to measure the changes in the deviation pattern between the native and mutation complexes (Fig 4). The native complex exhibited a deviation between ~0.2nm and ~0.4 nm until 30ns, and convergence was finally observed at the end of 50 ns, with an RMSD value of ~0.3nm (Fig 4). The R203C mutant complex exhibited the highest deviation at 10ns upto an RMSD value of ~0.5nm, which later converged to ~0.35nm at 20ns. An increase in the RMSD value up to ~0.45nm was observed at the end of the 50ns simulation. Meanwhile, the R131W mutant complex and native complex exhibited similar deviation patterns. The R131Q mutant complex exhibited a deviation between ~0.25 and ~0.35 that converged at 50ns, with an RMSD value of ~0.3nm (Fig 4). A root mean square fluctuation (RMSF) plot was constructed to measure the observed differences in fluctuation pattern between the native and mutant complexes. Overall, a greater fluctuation pattern was observed at terminal residues of the native and mutant complexes. We observed greater fluctuations in the R203C mutant complex (up to ~0.30nm), followed by the R131W and R131Q mutant complexes, which exhibited fluctuations of up to ~0.27nm. However, the native complex exhibited a minimal fluctuation of upto ~0.20nm (S4 Fig). The observed differences in the RMSD (Fig 4) were further validated by assessing the number of intermolecular hydrogen bonds formation between the protein and DNA in the native and mutant complexes (Fig 5). The average number of intermolecular hydrogen bonds formed between the native protein and DNA was 31.11. In mutant complexes, R203C showed the least number of intermolecular hydrogen bonds, with an average number of 20.075, whereas R131Q and R131W showed average numbers of intermolecular hydrogen bonds of 36.05 and 34.667, respectively (Fig 5). The least number of hydrogen bonds formation in the R203C mutant complex correlates with the higher deviation pattern observed in the RMSD analysis. The larger deviation explains the destabilizing effect because fewer hydrogen bonds were formed.

**Fig 6:**
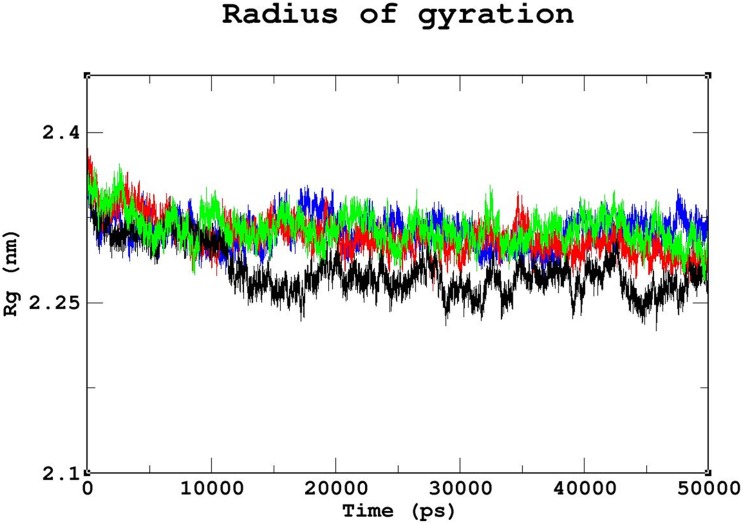
The radius of gyration analysis of native and mutant complexes. Color scheme: native (black), R131W (red), R131Q (green), and R203C (blue). The native complex exhibited the least Rg value, whereas the R203C mutant complex exhibited the highest Rg value. R131W and R131Q mutant complexes showed Rg values between the native and R203C mutant complex values.

The radius of gyration (Rg) was utilized to measure and understand the compactness of the protein complexes (Fig 6). The lowest Rg value of ~0.25nm was observed for the native complex. Among the three mutant complexes, the least compactness was observed for the R203C mutant complex, with an Rg value of~2.35nm, followed by the R131W and R131Q mutant complexes with Rg value ~0.3nm, which exhibited similar deviation patterns and convergence. The increased Rg value may also explain the loss of intermolecular hydrogen bonds as shown in Fig 5. The loss of compactness results from the reduction in the interaction pattern between the protein and DNA. Contact map analysis was conducted using g_mdmat to investigate this phenomenon. In the contact map analysis, a loss of contact in the anti-parallel beta sheet region was observed in all the mutant complexes, confirming the increased destabilizing effect upon mutation compared to the native (S5A–S5D Fig). Based on the results of the RMSD analysis, the intermolecular hydrogen bond formation, Rg values and contact map analysis, the R203C mutant complex exhibits the fewest interactions between the protein and DNA.

**Fig 7:**
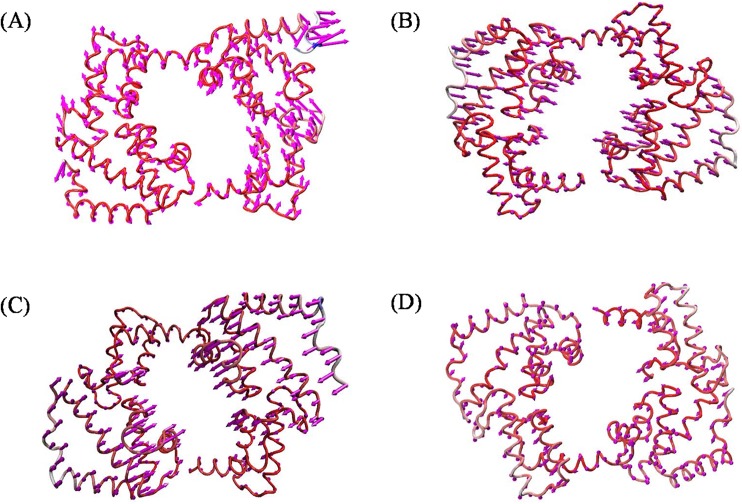
Porcupine plots of the PCA analysis depicting the movement and altitude of the C-alpha atoms throughout the 50 ns simulation in the native and mutant complexes. (A) Native, (B) R131W, (C) R131Q, and (D) R203C mutant complexes. The direction of the arrows indicates the direction of movement of the protein, and the length of the arrows indicates the magnitude of the motion. The R203C mutant complex showed the least difference in motion throughout the simulation compared to the native and other mutant complexes.

Essential dynamics analyses were elucidated to measure and understand the differences in motion between the native and mutant complexes. The g_anaeig and g_covar utilities from Gromacs were used to perform a PC analysis to reveal the changes in the motion patterns of the protein complexes (S6 Fig). A porcupine plot was drawn using VMD to explain this difference more accurately (Fig 7A–7D). The arrows present on the protein complex indicate the direction and magnitude of the motion. Motion differences were observed in all three mutant complexes compared to the native complex. Among the three mutant complexes, a larger deviation pattern was observed in R203C mutant complex. This deviation might be due to the unfolded structure as observed in the complexes, which was further analyzed using Free energy landscape (FEL) analysis.

**Fig 8:**
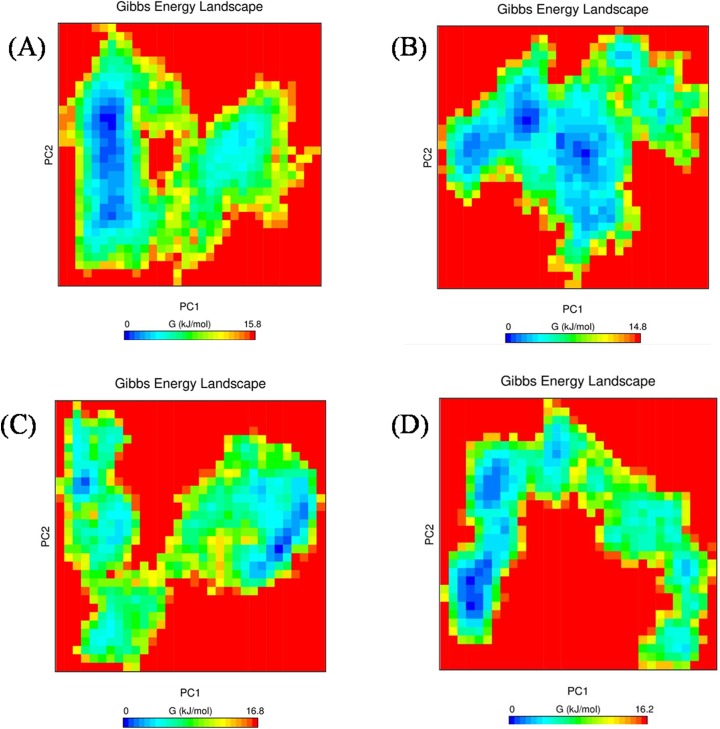
Free energy landscape between PC1 and PC2 of native and mutant complexes. (A) Native complex, (B) R131W complex, (C) R131Q complex, and (D) R203C complex.

The folding pattern of a protein is crucial for its proper function. FEL analyses helped us to determine whether the protein favors a folded or unfolded state, based on the obtained Gibbs free energy. Proteins with higher Gibbs free energy, which is shown as blue spots, favor an unfolded state, and a decrease in the Gibbs free energy, which is shown as red spots, favors a folded state in the FEL analysis. Among the three mutant complexes, R203C showed more blue spots (which correspond to increased Gibbs free energy) compared with the native protein (Fig 8A–8D),whereas the R131W and R131Q mutant complexes showed similar scattered red and blue spots and higher Gibbs free energy than the native molecule. Because the folding pattern of the protein directly influences its stability, a smaller number of hydrogen bonds coincide with decreased stability.

### Analysis of the electrostatic potential of the deleterious mutations

**Fig 9:**
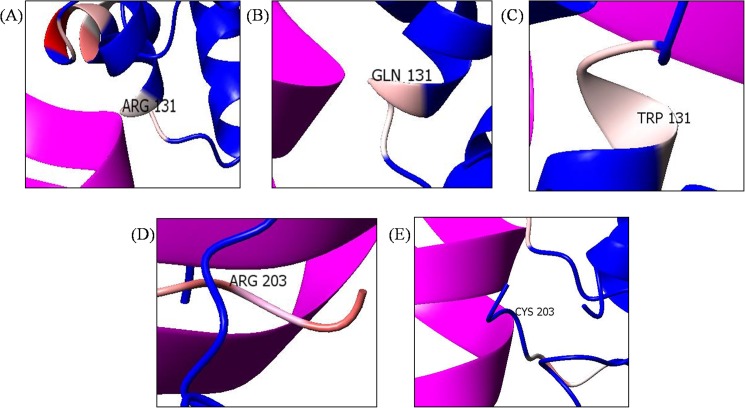
Electrostatic potential of the following residues: (A) native R131, (B) R131Q (C) R131W, (D) native R203, and (E) R203C. R131 in the native protein (A) was pale red in color and retained same color even after substituted with Tryptophan (B) and Glutamine (C). However, R203 in the native protein (D) was deeper red in color (larger positive charge), which subsequently changed to blue color (E) after substituted with Cysteine.

Most of the molecular interactions depend on the basic phenomenon of electrostatic potential. A comparative analysis of the electrostatic potential was conducted using CCP4mg. This visualization allowed us to understand the different electrostatic points potentials observed in the native and mutant complexes. In Fig 9A–9C, the arginine at position 131 had a negative charge (pale red region), which was maintained in the mutant complexes R131W and R131Q. However, the arginine at position R203 showed a deeper red color, which changed to a blue color when replaced with a cysteine (R203C mutant complex) (Fig 9D–9E). This change in color implies a shift in the electrostatic potential.

## Discussion

Missense mutations have long been known to inhibit protein activity, and tend to occur in DNA-binding regions of the protein affecting the DNA-binding affinity [18–20]. HNF1A is a transcription factor (DNA-binding protein) that regulates many liver, pancreas, and kidney-specific genes [48, 49]. The loss of function of this transcription factor leads to MODY (MODY3), which is most often known as monogenic diabetes [50]. The loss of the DNA-binding property of HNF1A due to the presence of a missense mutation at position 203 has received greater attention from researchers [12].

In our study, the missense mutations observed in HNF1A were subjected to rigorous pathogenic and stability analyses to identify the deleterious mutations and measure their predicted structural and functional impactson the protein. In recent years, several studies have highlighted the importance of *in silico* prediction methods in prioritizing deleterious mutations associated with simple and complex diseases [51]. Individual prediction tools have been developed using different algorithms, such as training datasets, machine learning methods and underlying principles (physio-chemical properties), to interpret their prediction scores as pathogenic/ disease/deleterious/destabilization vs benign/non-disease/benign/stabilization. A combined approach using diverse algorithms is believed to yielda single consensus prediction with improved accuracy [52, 53]. Therefore, we employed multiple prediction tools with diverse algorithms to identify eight highly pathogenic mutations (N127Y, R131Q, R131W, S142F, R159W, R200W, R203C, and R263C) that destabilize the protein. The primary focus of the present study is to analyze the effects of mutations located in the DNA-protein binding region of HNF1A (S7 Fig and S2 Table). Thus,we selected R131W, R131Q, and R203C mutants for further computational molecular studies. A powerful computational method, MD simulations [54–56] allowed us to explain the dynamic nature of the protein-DNA interaction and elucidate differences at an atomic level.

The stability and function of proteins are the two important interdependent properties that must be considered when studying protein structure, mutations that repress either property of a protein directly affect the other property [57, 58], and therefore, protein stability plays a significant role in preserving its function. In our study, we revealed that the effects of the R203C mutation on protein stability using MD. As shown by Yun *et al*. [2011], a higher RMSD is associated with reduced stability, consistent with our observation that the R203C mutant complex exhibited the largest deviation pattern among the three mutant complexes (Fig 4) [59], which was correlated with the reduction in the number of intermolecular hydrogen bonds formed in the R203C mutant complex compared with the native complex and other (R131W and R131Q) mutant complexes (Fig 5).

Studies of the evolutionary stability and mutational resistance of protein-coding genes have proven that arginine, leucine, and serine are the primary amino acids that affect protein stability in mutants [60]. Charge, hydrophobic-hydrophilic interactions, and binding patterns are vital for protein-DNA interactions [61]. The R131Q and R203C mutant complexes exhibited the greatest variations in hydrophilic-hydrophobic interactions compared with the native complex, as observed from the results obtained from CoCoMaps (S3 Table). When comparing the numbers of salt bridges formation in the complexes, we observed 13 salt bridges formation in the native, R131W and R131Q mutant complexes. Meanwhile, the R203C mutant complex involved only in five salt bridges formation. Salt bridges are known to experience thermal fluctuations that continuously break and re-form, and the flexibility of the protein governs the formation of the salt bridge (S4 Table). Therefore, these interactions are considered a vital factor governing the stability of the protein [62].

Arginine is a hydrophilic amino acid and is located in the exposed region (Fig 2). It has been reported that proteins have evolved to place arginine residues at their surfaces to stabilize their structures [63], and a favorable folding pattern was observed when arginine was present on the surface; conversely, a substitution with a cysteine residue resulted in reduced stability and increased Gibbs free energy, as observed in FEL analysis (Fig 8). The Gibbs free energy can explain the folding and unfolding patterns of proteins [64]. Additionally, the folding pattern correlates with protein structure and function [65]. Based on the results obtained from the RMSD analysis, the number of hydrogen bonds and the free energy landscape analysis, the R203C mutant complex showed differences in stability that further affected functionality. The changes in the Gibbs free energy in the R131W and R131Q mutant complexes were significant. When comparing the effects of the three mutants with the native protein, we conclusively define R203C mutation having greater effects on the protein.

Regarding the DNA interactions, DNA bases interact with more than one amino acid, and single interactions are rarely observed. In general, very few amino acids have base preferences, with the exceptions of arginine, serine, and histidine [66]. Several studies have emphasized the significant role of arginine in RNA-binding proteins and histone proteins in eukaryotic gene expression [67, 68]. Arginine is the most favored amino acid because of the length of its side chain, its capacity to interact in different conformations and, finally, its ability to produce good hydrogen-bonding geometries [69]. Consequently, substituting arginine with cysteine could lead to adverse effects. This phenomenon was observed in our study using contact maps, which showed a loss of contact at the anti-parallel beta sheet region in the R203C mutant complex (S5 Fig); however, a similar change was not observed in the R131W and R131Q mutant complexes. Generally, hydrogen bonds are formed in the anti-parallel beta sheets at 90° angles. The loss of contacts in this region leads to greater destabilization. The loss of contact might have occurred because cysteine, serine, and threonine have less affinity for DNA [63]. We observed a convincing change in theR203C mutant complex compared with the other mutant complexes. The elucidation of electrostatic potentials is a reliable way to predict the DNA-binding regions in DNA-binding proteins [70]. Following the substitution of arginine at position 203 with cysteine, an absolute charge difference was observed, unlike in the other two mutations (Fig 9). This finding further proves that substituting arginine with cysteine might interfere with DNA interactions. This finding was consistent with the results obtained from the molecular docking analysis (Table 3), where a smaller electrostatic energy was observed for the R203C mutant complex than for other mutant complexes.

In summary, the substitution of arginine (R) with cysteine (C) at position 203 decreases the affinity of the proteinfor DNA. Although the destabilizing effect of this mutant on the DNA-protein complex has been previously discussed [15, 71, 72], the present study used computational methods, such as MD, and *in silico* prediction methods to provide additional insights into the structure of this protein. It has been previously reported that the R131Q mutant retained the activity of the native protein, and R131W mutant showed 50% of the native protein activity [71, 72]. In the current analysis, we observed similar deviation patterns for the native, R131W and R131Q mutant complexes in the MD simulation analysis (Fig 4). Moreover, in the intermolecular hydrogen bond (Fig 5) and radius of gyration analyses (Fig 6), we observed similar patterns of decrease in the number of intermolecular hydrogen bonds and compactness in both the mutant (R131W and R131Q) complexes. Finally, when comparing the differences in electrostatic potential, we did not observe a significant change in the R131W and R131Q mutant complexes, whereas R203C mutant complex showed a shift in in electrostatic potential (Fig 9A–9C).Forthe R203C mutant complex, hydrogen bonds were observed with the base and sugar atoms, and hence, this position serves as a core element of the N-terminal arm in recognizing the minor groove, without imposing DNA specificity [15]. Substitution with cysteine at R203 (R203C) resulted in the formation of fewer intermolecular hydrogen bonds at the end of the simulation. In addition, the interactions with DNA are not specific and include electrostatic attractions (ionic salt bridges), which were also lost in the analyses of the electrostatic potential (Fig 9) and salt bridges (S4 Table). Recently, MD simulation studies have been the focus of biologists elucidating the various effects of mutations on proteins, protein-protein interactions, protein-DNA interactions and protein-ligand interactions [73–78]. In addition, this powerful method exhibited the best correlation with experimental studies [58, 79, 80]. Therefore, we hope the present application of a computational platform will explain the effects of mutations, further help in highlighting the potential economic benefit in reducing the cost of experimental analyses and the tedious process of mutational analysis.

## Conclusion

Transcription factors play pivotal roles in various cellular mechanisms, including regulation of cell function, growth, and differentiation, The detailed molecular structure analysis of the HNF1A presented here emphasizes the importance of the two arginine positions R131 and R203 for the HNF1A stability and function, the mutants R131Q and R203C were shown to be deleterious for the protein function and compromise its binding affinity, however our data suggested that the R203C mutant was the most deleterious leading to the loss of the protein binding affinity. The understanding of the detailed molecular structure of the mutations in HNF1A that cause MODY3 is expected to serve as a platform for developing therapeutic approaches for patients with MODY3 and drug discovery for treating diabetes, and maycreate a path toward personalized medicine for diabetic patients.

## Materials and methods

### Dataset collection

The mutations associated with the *HNF1A* gene were retrieved from the NCBI dbSNP (http://www.ncbi.nlm.nih.gov/snp/), UniProt (http://www.uniprot.org/), and HGMD databases (http://www.hgmd.org/) to understand their functional annotation. Further information about the impact of the mutations and their disease associations was collected based on *in vivo* and *in vitro* experiments recorded in OMIM (http://www.ncbi.nlm.nih.gov/omim/), PubMed (http://www.ncbi.nlm.nih.gov/PubMed/), and UniProtKB (http://www.uniprot.org/).

### Analysis of pathogenic mutations

A set of 10 *in silico* tools was used to predict the pathogenicity of each mutation collected from the above mentioned databases. SIFT (Sorting Intolerant from Tolerant) (http://sift.bii.a-star.edu.sg/) [36] and SNPs&GO (http://snps-and-go.biocomp.unibo.it/snps-and-go/) predict the impacts of coding mutations on protein function [38]. PolyPhen-2 (Polymorphism Phenotyping v2) (http://genetics.bwh.harvard.edu/pph2/) predicts the influence of a substitution on the structure and function of a protein using its physical properties. [34]. HANSA (http://www.cdfd.org.in/HANSA/) [35] and PhD-SNP (http://snps.biofold.org/phd-snp/) [29] are support vector machine (SVM)-based methods used to classify mutations into disease-causing or benign mutations using six position-specific probabilities. Fathmm (http://fathmm.biocompute.org.uk/) is an HMM (Hidden Markov Model) algorithm-based tool that is capable of predicting the functional consequences of coding missense mutations [30]. PANTHER (http://www.pantherdb.org/about.jsp) [37] actually predicts the pathogenicity of a mutation based on the evolutionary pattern. The biophysical analysis of the effect of the mutation was calculated using the Align GV-GD tool (http://agvgd.iarc.fr/agvgd_input.php) [31]. SNAP is another highly validated tool that predicts a mutation based on linkage disequilibrium (LD) (http://www.broad.mit.edu/mpg/snap/) [32]. The PONP2 (http://structure.bmc.lu.se/PON-P2/) tool is a machine learning approach that predicts the harmful effects of mutations by utilizing the evolutionary sequences and the biochemical and physical properties of the protein [33].

### Protein stability tools

The relationships between the mutations and their effects on protein stability were calculated using three *in silico* prediction tools. I-Mutant 2.0 (http://folding.biofold.org/i-mutant/i-mutant2.0.html) [34], based on SVM, calculates the direction of the changes inprotein stability and the energy values associated (ΔΔG) with predicting stability. If the variations do not reduce stability, then they are designated as neutral. MUpro incorporates both SVM and neural network approaches to predict the differences in stability caused by an amino acid substitution. The output of this tool describes the changes inenergy (ΔΔG) due to the substitution of the amino acid, with a confidence score that ranges from -1 and 1. A variation with a score of less than 0 decreases the stability of the protein (www.igb.uci.edu/servers/servers.html) [35]. In addition, the prediction in the DUET is based on the change in the folding free energy (http://bleoberis.bioc.cam.ac.uk/duet/stability) [36]. This server further integrates two other methods to generate its prediction (SDM and mCSM).

### Analysis of protein-DNA interactions

Transcription factors are the set of proteins that bind to a specific region of the DNA to up-regulate or down-regulate a specific transcription process. The binding of the protein to the DNA depends on highly specific regions or residues present in the protein. The BindN+ tool was used to identify the protein-DNA interaction. BindN+ (http://bioinfo.ggc.org/bindn+/) is an SVM-based approach that utilizes three features of the sequence, namely, the side chain pKa, hydrophobicity index, and molecular mass of the given amino acid, to make its prediction. The input for the tool is in FASTA format, and the output is depicted as positive (+) and negative (-). The positive (+) sign represents the presence of binding sites, and the negative (-) sign represents the absence of binding sites [42]. The obtained results were further compared with the binding sites available in PDBsum to validate the residues in the protein that interacted with the DNA. PDBsum (http://www.biochem.ucl.ac.uk/bsm/pdbsum) aims to provide a summary of the molecules that are confined in the PDB entry [81].

### Sequence conservation

The presence of conserved regions among the homologous sequences of the protein was investigated using the ConSurf web server (http://consurf.tau.ac.il/). This web-based algorithm predicts the functionally important regions of a protein by estimating the degree of conservation of amino acid sites based on homology [82]. The grades range from 1 to 9 represent the extent of conservation of the amino acid throughout evolution. Therefore, grade of 9 represent the most highly conserved residue, and the numbers descend to 1, which represent the least conserved region. This tool analyzes the conservation at the nucleotide andamino acid levels.

### Structural analysis

The UniProtKB (http://www.uniprot.org/) database describes the protein and provides a detailed explanation and the FASTA sequence of the protein. The sequence of the human HNF1A (P20823) protein with a length of 631 amino acids was retrieved. The transcript with the ref sequence NP_001293108 was used in the study (http://www.ncbi.nlm.nih.gov/protein/NP_001293108.1). The X-ray structure (PDB: 1IC8) with a resolution of 2.8 Å was obtained from the PDB database [12]. The DeepView (Swiss PDB Viewer) visualization and structural analysis tool was used to identify the corresponding positions of the mutations [83]. For a better analysis of the mutants, the mutation proteins were docked with the DNA using the HADDOCK server [44], and the obtained complexes were used for further analysis.

### Interaction analysis

CoCoMaps (bioCOmplexes Contact MAPS) visualizes the interface between the chains as presented in the X-ray or NMR structures [43]. The input file is a structure file in PDB format (http://www.molnac.unisa.it/BioTools/cocomaps). Along with hydrogen bond analysis, we also predicted the number of salt bridges that were formed in the molecules using the ESBRI server (http://bioinformatica.isa.cnr.it/ESBRI/). The server works on a CGI script written in Perl language that elucidates the interactions present between the oppositely charged groups and recognizes at least one Asp or Glu side-chain carboxyl oxygen atom and one side-chain nitrogen atom of Arg, Lys or His within a distance of 4.0 Å [44].

### Molecular dynamics simulation

MD simulations and energy minimization were conducted using the Gromacs 4.6.3 software package with the force field CHARMM 27 [84]. The native structure of the protein was obtained from the Protein Databank (PDB: 1IC8) and used for further studies. The native and deleterious mutation complexes were subjected to MD simulations. The protein atoms were placed in a cubic box with the 0.9-nm simple point charge (SPC), and further periodic boundary conditions were optimized to perform the simulations. The system was solvated, and sufficient number of sodium and chloride ions was used to neutralize the charge of the system. Energy minimization was conducted using the steepest descent method to provide a stable conformation. The temperature was maintained at 300K. Canonical Ensembles (NVT) and Isobaric-Isothermal Ensembles (NPT) were performed (each 50,000 steps). Following the equilibration procedures, MD simulations were conducted for the native and mutation complexes for 50 ns. Three 50 ns runs for each system (native-DNA complex and mutant-DNA complexes) were performed. The resulting trajectory files of the simulations were analyzed using various parameters available in Gromacs utilities. g_rms, g_rmsf, g_hbond, g_mdmat, g_gyrate, g_covar, g_anaieg, and g_sham were used to calculate the RMSD, RMSF, H-bond interactions, interacting residues, radius of gyration, PCA and free energy landscape of the protein-DNA structures. The resulting files for these parameters were analyzed using the Graphing, Advanced Computation, and Exploration (XMGRACE) program. A porcupine plot was drawn using VMD [85], and an electrostatic potential analysis was conducted using CCP4mg [86].

## Supporting information

S1 Fig (A) Workflow explaining the different databases used to collect the protein and mutation information. (B)Workflow explaining the process used to select the deleterious mutations (‘N’ denotes number of missense mutations).
(TIF)

S2 Fig Percentage of deleterious mutations identified using different in silico pathogenicity prediction tools. (TIF)

S3 Fig The RMSD plot of run1 and (b) RMSD plot of run3. Color scheme: native complex (black), R131W complex (red), R 131Q complex (green), and R203C complex (blue).
(TIF)

S4 Fig The RMSF plot of the native and R131W, R131Q, and R203C mutant complexes. Color scheme: native complex (black), R131W complex (red), R131Q complex (green), and R203C complex (blue).
(TIF)

S5 Fig Contact maps analysis. (A) native, (B) R131W, (C) R131Q, and (D) R203C mutant complexes.
(TIF)

S6 Fig Principle Component Analysis of the native and R131W, R131Q, and R203C mutant complexes. (TIF)

S7 Fig DNA-interacting residues in the HNF1A protein obtained using PDBsum. (TIF)

S1 Table SNP analysis tools used to predict the pathogenicity of the missense mutations in HNF1A. (DOCX)

S2 Table Comparative analysis of the DNA-binding sites of HNF1A missense mutations predicted by PDBsum and BindN+. (DOCX)

S3 Table Interactions observed between the DNA and the protein in native and R131W, R131Q, and R203C mutant complexes. (DOCX)

S4 Table Number of salt bridges formation in the native and mutant (R131W, R131Q, and R203C) complexes. (DOCX)
