# Supplementary figures and images for: Determining the role of missense mutations in the POU domain of HNF1A that reduce the DNA-binding affinity: A computational approach
Source: PLoS One. 2017 Apr 14;12(4):e0174953. doi: 10.1371/journal.pone.0174953 (PMC5391926; doi:10.1371/journal.pone.0174953)

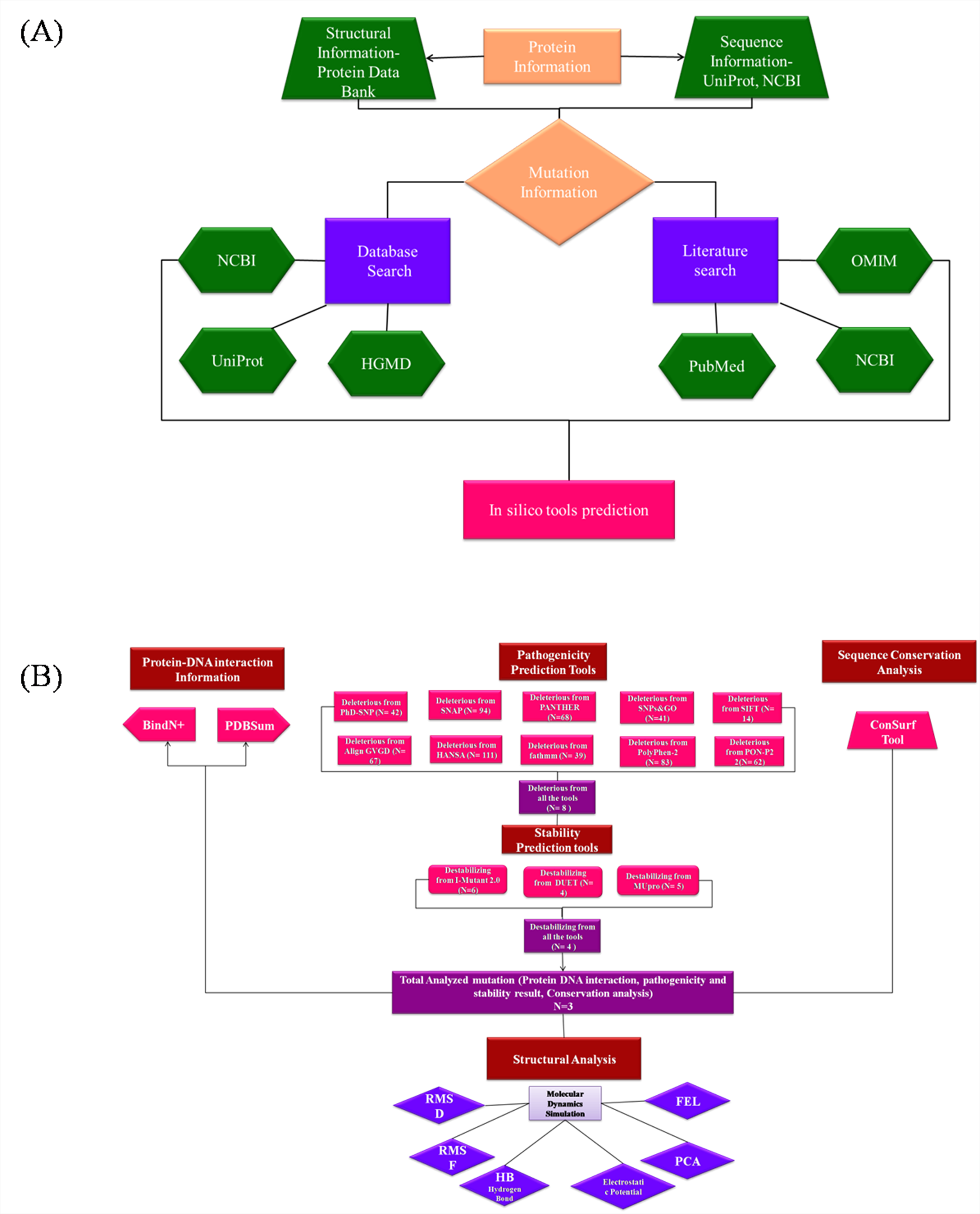

Supplement: S1 Fig — (A) Workflow explaining the different databases used to collect the protein and mutation information. (B)Workflow explaining the process used to select the deleterious mutations (‘N’ denotes number of missense mutations). (TIF) [file pone.0174953.s001.tif]

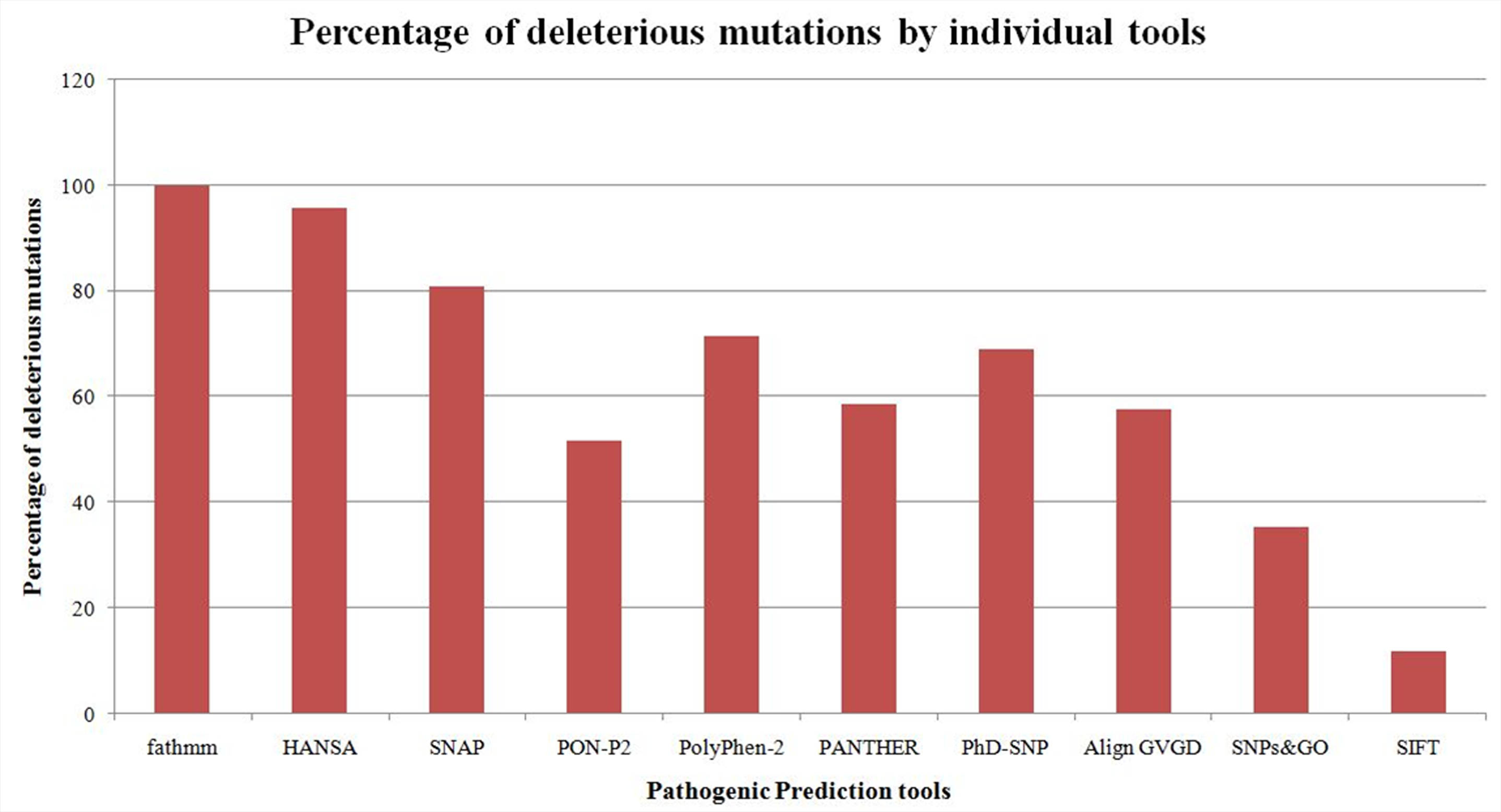

Supplement: S2 Fig — (TIF) [file pone.0174953.s002.tif]

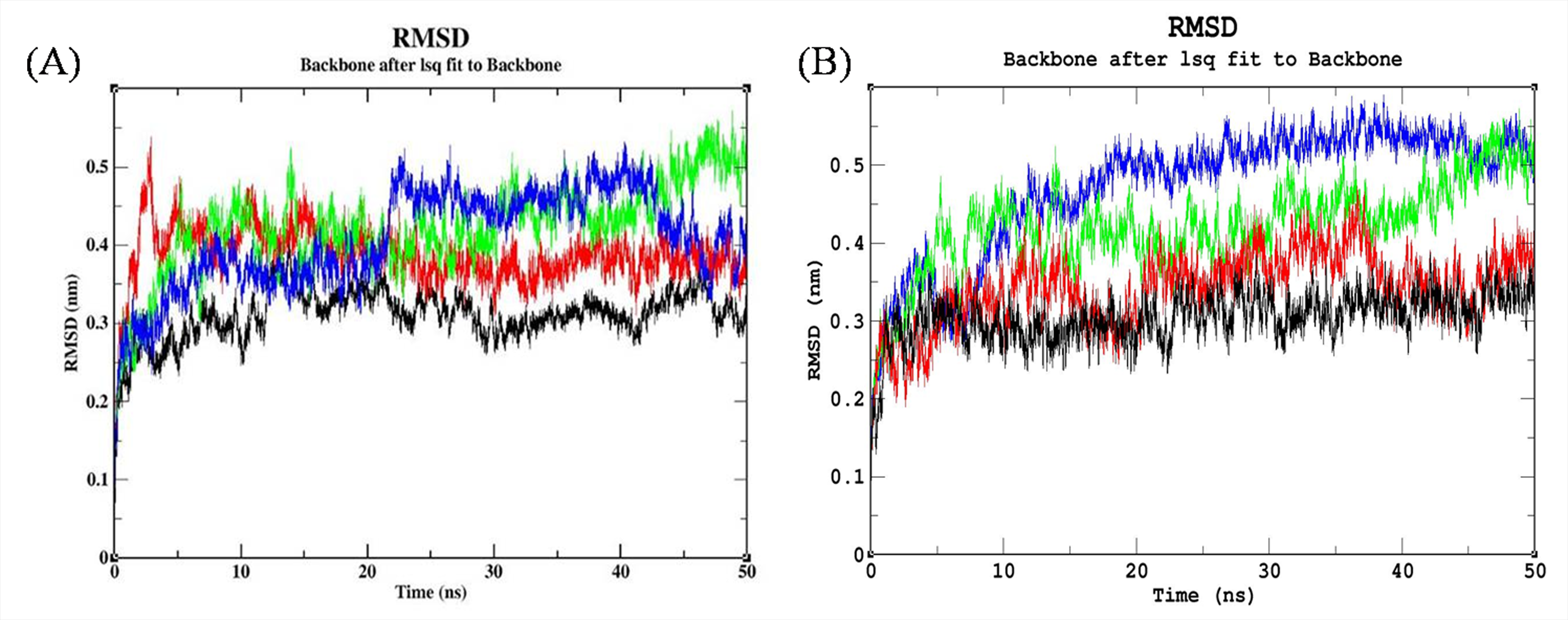

Supplement: S3 Fig — Color scheme: native complex (black), R131W complex (red), R 131Q complex (green), and R203C complex (blue). (TIF) [file pone.0174953.s003.tif]

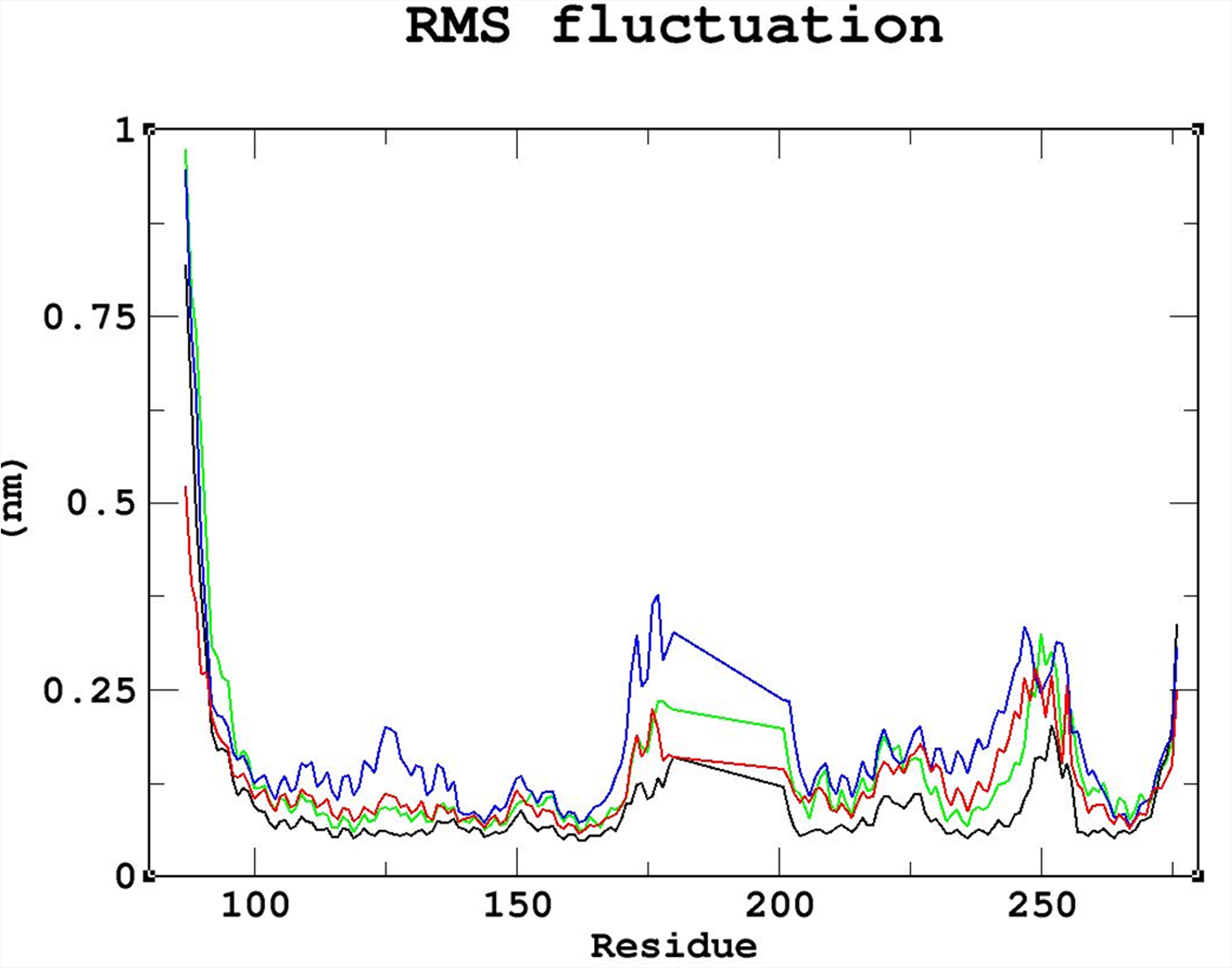

Supplement: S4 Fig — Color scheme: native complex (black), R131W complex (red), R131Q complex (green), and R203C complex (blue). (TIF) [file pone.0174953.s004.tif]

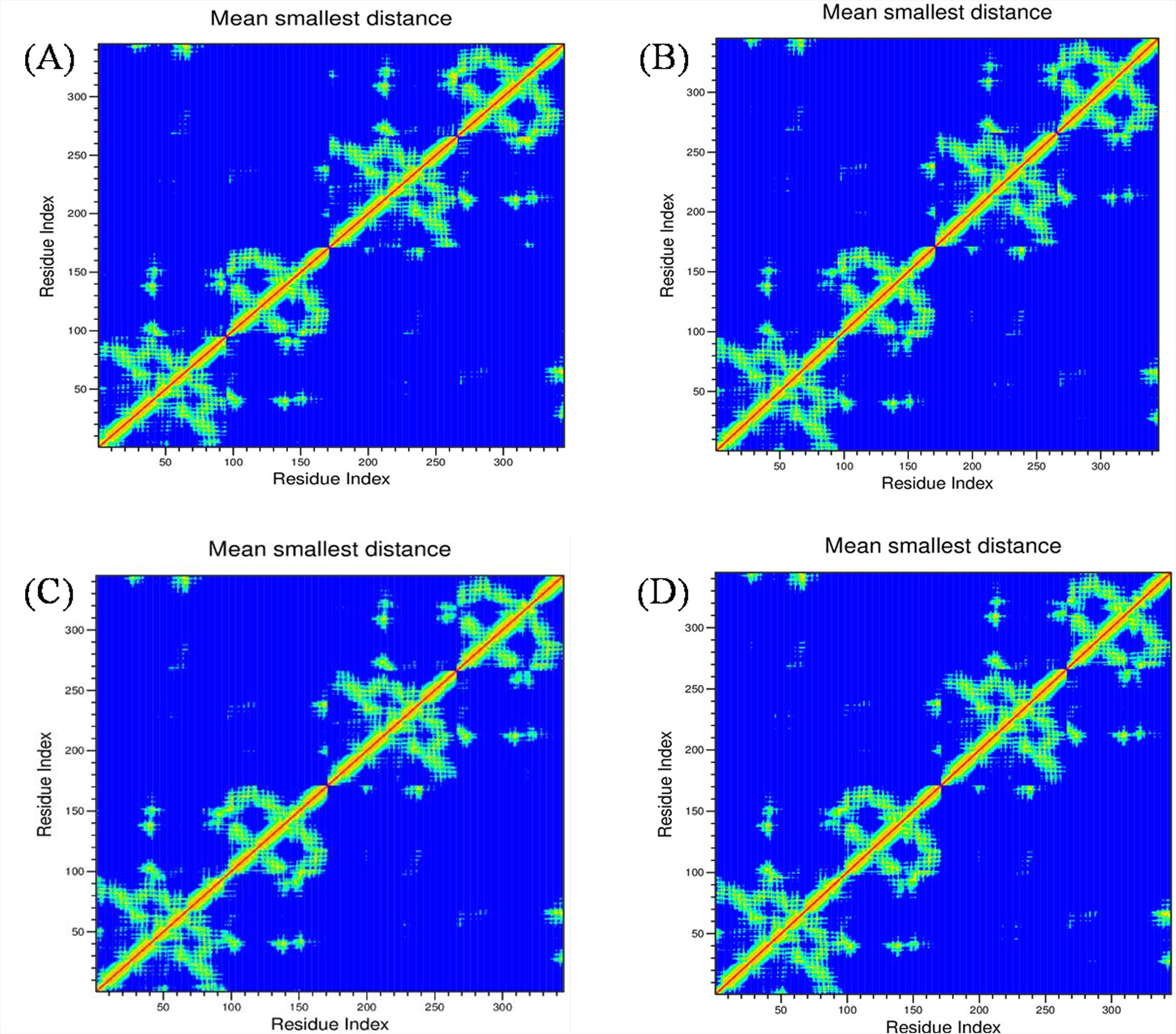

Supplement: S5 Fig — (A) native, (B) R131W, (C) R131Q, and (D) R203C mutant complexes. (TIF) [file pone.0174953.s005.tif]

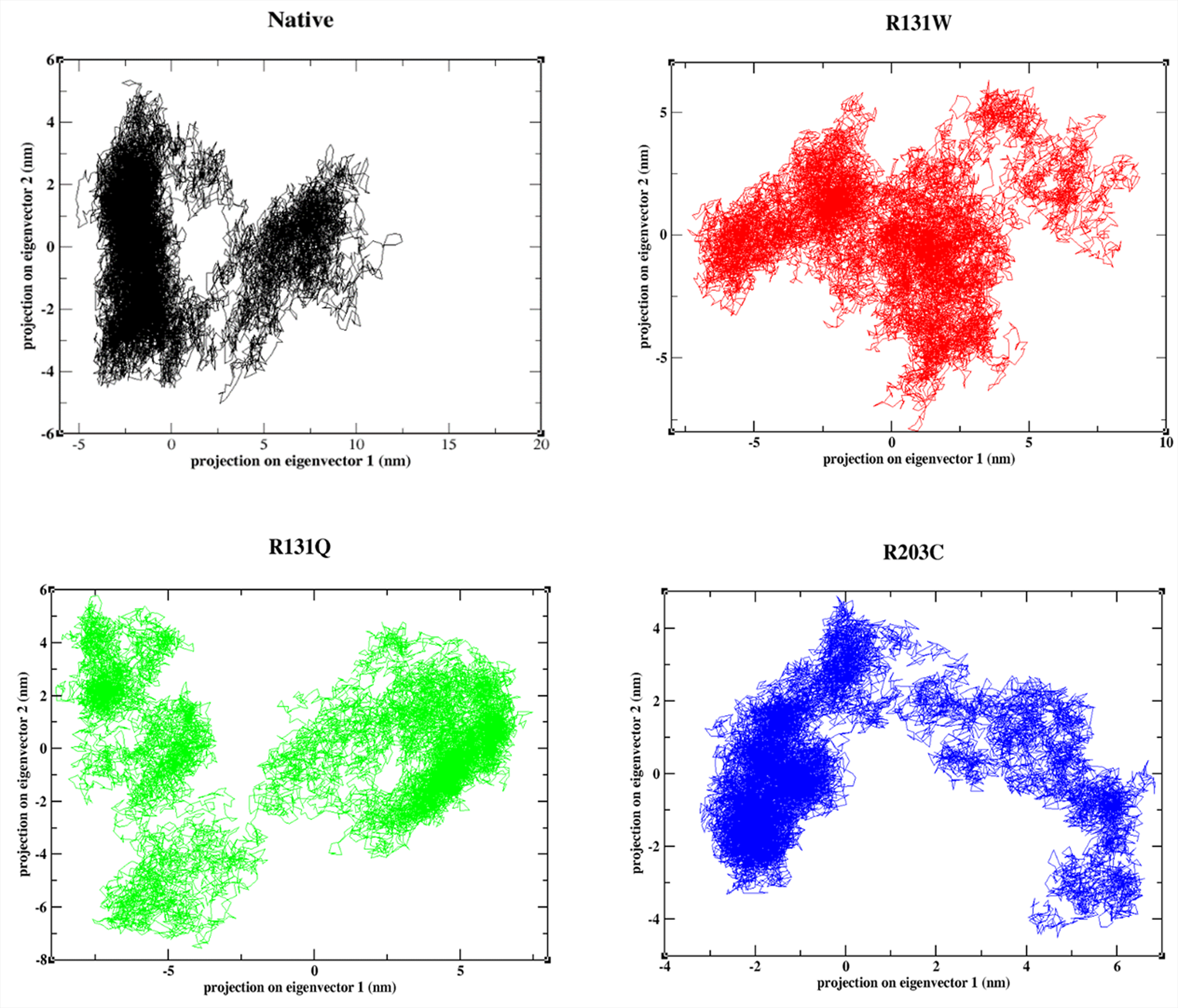

Supplement: S6 Fig — (TIF) [file pone.0174953.s006.tif]

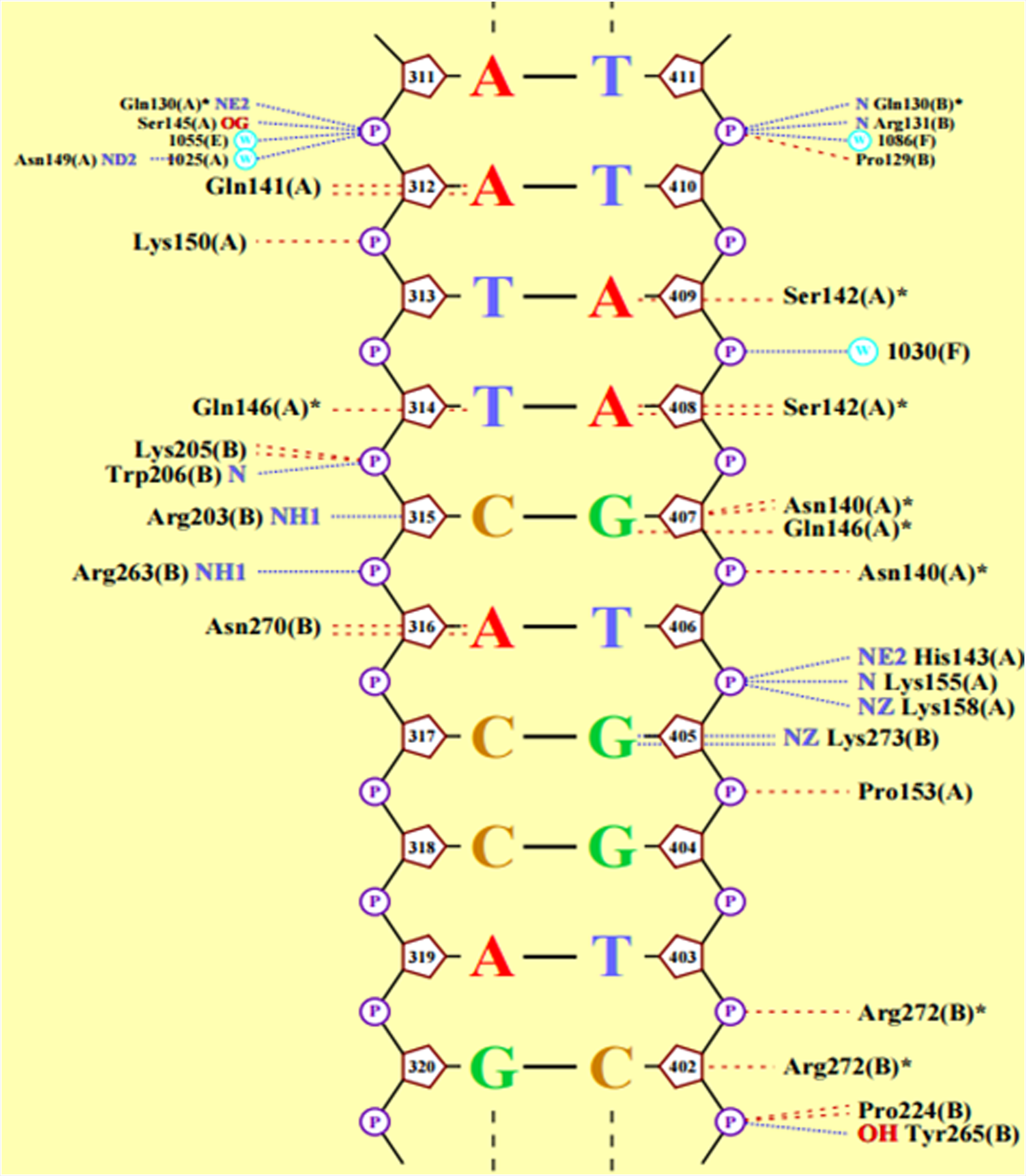

Supplement: S7 Fig — (TIF) [file pone.0174953.s007.tif]
