# Supplementary material for: Determining the role of missense mutations in the POU domain of HNF1A that reduce the DNA-binding affinity: A computational approach
Source: PLoS One. 2017 Apr 14;12(4):e0174953. doi: 10.1371/journal.pone.0174953 (PMC5391926; doi:10.1371/journal.pone.0174953)
Supplement: S1 Table — (DOCX) [file pone.0174953.s008.docx]

**S1 Table.** SNP analysis tools used to predict the pathogenicity of the missense mutations in HNF1A

| **HGMD/UniProt/dbSNP IDs** | **Mutations** | **PhD-SNP** | **Align GVGD** | **SNAP** | **PON-P2** | **PolyPhen- 2** | **HANSA** | **SIFT** | **PANTHER** | **SNPs&GO** | **fathmm** |
| --- | --- | --- | --- | --- | --- | --- | --- | --- | --- | --- | --- |
| CM082819 | M1L | N | **C0** | **N** | N | N | N | 0.6 | D | N | -3.84 |
| rs193922592 | M1V | N | C15 | NN | UN | PoD | D | 0 | UC | N | -5.25 |
| rs188016232 | L5P | D | C65 | NN | P | PrD | D | 0.06 | D | N | -3.94 |
| CM085467 | S6N | D | C45 | NN | P | PrD | N | 0.5 | D | N | -4.44 |
| CM082862 | L8M | N | C0 | N | N | B | N | 0.8 | N | D | -3.33 |
| CM064305 | Q9P | D | C65 | NN | N | UN | N | 0.7 | N | N | -4.34 |
| CM064303 | T10M | N | C65 | N | P | B | N | 0.06 | N | N | -3.87 |
| VAR_010537 | L12H | D | C65 | NN | UN | PrD | D | 0 | D | N | -5.87 |
| CM052920 | L12F | N | C15 | N | UN | PoD | N | 0 | D | N | -5.55 |
| CM082857 | A14V | N | C65 | N | N | PrD | D | 0.05 | D | D | -3.45 |
| CM1111339 | L17H | D | C65 | NN | N | UN | N | 0.65 | D | D | -6.42 |
| CM082861 | L17V | N | C25 | NN | N | B | N | 0.87 | N | D | -3.88 |
| VAR_012483 | G20R | D | C65 | NN | UN | PrD | D | 0 | D | D | -6.17 |
| CM082861 | G20A | D | C55 | NN | P | B | N | 0 | D | D | -3.44 |
| CM052000 | A25P | N | C25 | N | UN | PoD | D | 0.56 | N | N | -6.54 |
| VAR_007905/rs1169288 | I27L | N | C0 | N | UN | B | D | 0.4 | N | N | -4.99 |
| CM082811 | I27T | N | C65 | N | UN | PrD | D | 0.3 | N | N | -5.43 |
| CM082812 | I27S | D | C65 | N | N | B | N | 0.39 | D | N | -6.57 |
| VAR_010538/rs137853247 | G31D | N | C65 | NN | UN | B | D | 0.55 | N | N | -4.85 |
| CM082832 | P33L | N | C65 | NN | P | PoD | N | 0.29 | D | D | -3.44 |
| rs139742280 | G34R | N | C0 | N | UN | B | D | 0.45 | N | N | -4.88 |
| rs373180062 | G47R | N | C65 | NN | UN | B | D | 0.45 | N | N | -4.9 |
| CM030524 | G47Q | N | C65 | N | P | PoD | N | 0.45 | D | D | -4.8 |
| CM109043 | E48D | D | C35 | N | P | PrD | N | 0 | N | D | -5.45 |
| VAR_010539 | E48K | N | C55 | N | UN | B | D | 0.64 | N | N | -4.88 |
| rs142318174 | G52A | N | C55 | N | UN | B | N | 0.75 | N | N | -4.93 |
| rs377129682 | N62S | N | C45 | N | UN | Pr D | D | 1 | N | N | -4.95 |
| rs188085301 | R68Q | N | C35 | NN | UN | B | D | 0.38 | N | N | -4.96 |
| CM082876 | R68W | N | C65 | N | N | B | N | 0.29 | N | N | -3.44 |
| rs143753579 | E79V | N | C65 | NN | UN | Po D | D | 0.17 | D | N | -5.16 |
| rs76845985 | D80Y | D | C65 | NN | UN | Pr D | D | 0. 01 | N | N | -5.05 |
| rs200442958 | L86H | D | C65 | N | P | Pr D | D | 0. 54 | D | N | -5.29 |
| rs193922593 | P94L | N | C65 | N | UN | B | D | 0.39 | D | N | -4.97 |
| rs374794304/CM971442 | A97V | N | C65 | NN | P | Po D | D | 0. 15 | D | N | -5.04 |
| VAR_010540 | A98V | N | C65 | N | UN | B | D | 0.49 | D | N | -5.21 |
| rs367839639 | V103M | N | C15 | NN | UN | Pr D | D | 0.1 | N | N | -4.98 |
| VAR_010541/CM971443 | L107R | D | C65 | NN | UN | Pr D | D | 0.16 | D | D | -5.61 |
| CM993621 | L107I | N | C0 | NN | P | B | D | 0.03 | D | D | -4.55 |
| CM082838 | L107V | N | C25 | N | P | PrD | N | 0.29 | N | D | -3.7 |
| rs137853243/CM004772 | P112L | D | C65 | N | P | Pr D | D | 0.01 | N | N | -5.37 |
| CM093340 | R114C | D | C65 | NN | P | PoD | N | 0.03 | D | D | -4.55 |
| rs139016696 | R114H | N | C25 | NN | UN | B | D | 0.13 | N | N | -5.12 |
| CM064316 | V115L | N | C25 | N | N | B | D | 0.39 | D | N | -4.56 |
| CM082865 | A116T | D | C55 | NN | N | PrD | N | 0.45 | N | D | -5.45 |
| CM015315 | A116V | N | C65 | N | N | B | N | 0.03 | D | D | -344 |
| VAR_010542/CM991165 | K117E | N | C55 | NN | P | Pr D | D | 0.97 | N | N | -5.44 |
| VAR_003756/rs137853237 | Y122C | D | C65 | NN | P | Pr D | D | 0.67 | D | D | -5.94 |
| CM085468 | L123P | D | C65 | NN | N | PoD | N | 0.03 | D | N | -5.44 |
| CM082846 | L123R | D | C65 | NN | UN | PoD | N | 0.23 | D | D | -4.32 |
| VAR_033088 | N127Y | D | C65 | NN | P | Pr D | D | 0.33 | D | D | -5.72 |
| VAR_010543/CM971445 | I128N | D | C65 | NN | P | Pr D | D | 0.67 | D | D | -5.54 |
| VAR_010544/CM971446 | P129T | D | C35 | NN | P | Pr D | D | 0.78 | D | N | -6.21 |
| CM067042 | Q130H | N | C15 | N | N | N | N | 0.6 | N | N | -3.44 |
| VAR_010545 | R131Q | D | C65 | NN | P | Pr D | D | 0. 01 | D | D | -5.86 |
| VAR_010546/rs137853244 | R131W | D | C65 | NN | P | Pr D | D | 0.02 | D | D | -5.91 |
| CM082851 | G132N | D | C15 | NN | P | N | D | 0.5 | N | N | -4.33 |
| CM020514 | G132K | D | C25 | N | P | N | N | 0.7 | N | D | -4.45 |
| VAR_010547 | V133M | D | C15 | NN | P | Pr D | D | 0.04 | D | N | -5.19 |
| CM082870 | V133L | N | C25 | N | N | N | D | 0.06 | D | D | -6.44 |
| CM082843 | D135N | N | C15 | NN | N | N | D | 0.04 | N | D | -4.55 |
| CM082808 | T137S | D | C55 | NN | UN | PrD | N | 0.6 | N | N | -3.23 |
| CM082878 | G138S | D | C55 | N | N | PoD | D | 0.4 | N | N | -2.33 |
| VAR_003757/CM971448 | S142F | D | C65 | NN | P | Pr D | D | 0.3 | D | D | -5.74 |
| VAR_010548/CM971449 | H143Y | D | C65 | NN | P | Pr D | D | 1 | D | N | -5.78 |
| rs193922597 | H147Q | N | C15 | NN | P | Pr D | D | 0.51 | D | D | -5.79 |
| CM082837 | L148I | N | C0 | N | UN | B | N | 0.77 | N | D | -4.56 |
| CM082802 | N149K | D | C65 | NN | P | PoD | D | 0.03 | D | N | -6.45 |
| CM064299 | K150N | D | C65 | NN | P | PoD | D | 0.3 | D | D | -5.67 |
| CM082814 | M154R | D | C65 | NN | P | PoD | N | 0.03 | N | N | -5.66 |
| CM082820 | M154V | N | C15 | N | N | B | N | 1 | N | D | -4.56 |
| CM082813 | M154T | D | C65 | NN | UN | PoD | N | 0.04 | D | D | -3.44 |
| rs185620161 | K155R | N | C25 | NN | P | Pr D | D | 0.25 | D | N | -5.68 |
| rs150513055 | T156M | N | C65 | NN | P | Pr D | D | 0.13 | D | N | -5.03 |
| VAR_010549/CM991166 | K158N | D | C65 | N | P | Pr D | D | 0.21 | D | D- | -5.75 |
| VAR_003758/CM971450 | R159Q | D | C35 | NN | P | Pr D | D | 0. 00 | D | D | -5.86 |
| VAR_010550/CM981896 | R159W | D | C65 | NN | P | Pr D | D | 0.02 | D | D | -5.91 |
| VAR_010551/rs201095611/CM981897 | A161T | D | C55 | NN | P | Pr D | D | 0.23 | D | N | -5.14 |
| CM112115 | L162P | D | C65 | NN | UN | PoD | D | 0.34 | N | D | -3.44 |
| VAR_033089 | W165C | D | C65 | NN | P | Pr D | D | 1 | D | D | -6.17 |
| rs371759652 | V167I | N | C25 | NN | UN | Po D | D | 0.19 | N | N | -4.99 |
| rs377110124/CM064306 | R168P | N | C25 | NN | UN | Pr D | D | 0.12 | N | N | -5.26 |
| CM093342 | R171G | D | C65 | NN | P | N | D | 0.23 | N | N | -3.67 |
| CM082869 | V173M | N | C15 | N | P | D | N | 0.56 | D | D | -4.55 |
| rs201934320 | A174V | N | C65 | NN | UN | B | D | 0.29 | N | N | -5.02 |
| rs147400498 | Q175R | N | C35 | N | UN | B | N | 0.6 | N | N | -4.88 |
| VAR_010552/CM971452 | G191D | N | C65 | NN | P | B | D | 0.61 | D | N | -5.5 |
| rs139712739/CM082803 | T196A | N | C55 | N | UN | B | D | 0.82 | N | N | -3.68 |
| VAR_063069/rs193922598/CM981899 | R200W | D | C65 | NN | P | Pr D | D | 0.34 | D | D | -4.35 |
| CM981898 | R200Q | D | C35 | NN | P | PoD | N | 1 | N | N | -3.78 |
| CM030525 | R200G | D | C65 | NN |  | PrD | N | 0.19 | D | N | -2.33 |
| VAR_010554/CM991167 | R203C | D | C65 | NN | P | PrD | D | 0.2 | D | D | -5.53 |
| VAR_012484/CM993816 | R203H | D | C25 | NN | P | PrD | D | 0.02 | D | D | -5.51 |
| CM109044 | R203G | D | C15 | NN | P | B | D | 0.29 | D | D | -5.52 |
| VAR_010555/CM971453 | K205Q | N | C45 | NN | P | B | D | 0.26 | D | N | -3.95 |
| VAR_033090 | W206C | D | C65 | NN | P | PrD | D | 0.67 | D | D | -3.89 |
| VAR_033091 | W206L | D | C55 | NN | P | PoD | D | 0.67 | D | D | -3.76 |
| CM064312 | G207D | D | C65 | NN | P | PrD | D | 0.29 | N | N | -3.44 |
| CM087902 | I213T | D | C65 | NN | UN | PrD | D | 0.29 | N | D | -4.56 |
| CM082854 | A217G | D | C55 | NN | UN | B | D | 0.77 | D | D | -4.35 |
| CM003341 | Y218C | D | C65 | NN | UN | PrD | N | 0.32 | D | N | -5.44 |
| rs193922600 | P224S | D | C65 | NN | UN | PrD | D | 0.12 | D | D | -5.45 |
| CM082816 | K226E | D | C55 | N | P | B | D | 0.67 | N | D | -6.45 |
| CM082864 | E228K | D | C55 | NN | P | B | D | 0.74 | N | N | -6.33 |
| VAR_010556/CM971454 | R229Q | D | C35 | NN | P | PrD | D | 0.16 | D | D | -4.39 |
| CM002864 | R229P | D | C65 | NN | UN | PrD | N | 0.21 | D | D | -5.45 |
| CM023587 | V233L | N | C25 | N | UN | B | N | 0.87 | D | N | -7.67 |
| CM109039 | E235G | N | C65 | NN | P | PoD | D | 0.85 | D | N | -2.34 |
| VAR_033092 | N237S | N | C45 | NN | P | B | D | 0.67 | D | D | -3.83 |
| CM082866 | A239T | D | C55 | N | UN | PoD | D | 1 | D | D | -2.33 |
| CM023588 | A239V | N | C65 | N | UN | PoD | N | 0.65 | D | D | -3.12 |
| VAR_010557/CM971456 | C241G | D | C65 | NN | UN | PrD | D | 0.4 | D | D | -3.68 |
| CM981900 | C241R | D | C65 | NN | Un | PrD | N | 0.34 | N | D | -5.45 |
| CM971456 | C241G | D | C65 | NN | UN | PrD | N | 0.32 | N | D | -5.45 |
| rs193922602 | R244I | N | C65 | NN | P | PrD | D | 0.41 | D | D | -3.76 |
| VAR_033093 | R244G | N | C65 | NN | P | PrD | D | 0.41 | D | D | -3.69 |
| CM093344 | G245R | D | C65 | NN | P | PrD | N | 0.21 | N | D | -4.33 |
| CM064313 | V246L | N | C25 | NN | P | PoD | N | 0.29 | D | D | -4.32 |
| CM082871 | V246G | D | C65 | NN | P | PrD | N | 0.12 | D | D | -4.65 |
| rs193922603 | G245V | D | C65 | NN | P | PrD | D | 0.65 | D | D | -3.72 |
| CM087210 | S249P | D | C65 | N | Un | PoD | N | 0.29 | D | N | -3.45 |
| VAR_033094 | Q250P | N | C65 | NN | P | PrD | D | 0.35 | D | D | -3.83 |
| VAR_010558/CM020515 | L254M | N | C0 | NN | P | PrD | D | 0.15 | D | N | -4.06 |
| CM082868 | G255S | D | C55 | N | UN | PoD | D | 0.29 | N | D | -4.66 |
| CM030526 | S256T | D | C55 | NN | UN | PoD | D | 0.78 | D | N | -6.54 |
| CM087211 | N257T | D | C55 | NN | UN | PoD | N | 0.56 | D | D | -8.65 |
| VAR_010559/CM002111 | V259D | D | C65 | NN | P | PrD | D | 0.66 | D | D | -4.02 |
| VAR_010560/CM971457 | T260M | D | C65 | NN | P | PrD | D | 0.76 | D | D | -3.93 |
| CM064311 | G261K | N | C15 | N | N | B | D | 0.66 | D | N | -2.33 |
| CM031753 | R263H | N | C25 | N | N | B | N | 0.36 | N | D | -5.45 |
| VAR_010561/CM971458 | R263C | D | C65 | NN | P | PrD | D | 0.24 | D | D | -4.99 |
| rs193922604 | V264F | D | C45 | NN | P | PrD | D | 0.39 | D | D | -6.62 |
| rs193922605 | F268S | D | C65 | NN | P | PrD | D | 0.87 | D | D | -6.62 |
| CM082804 | N266K | D | C65 | NN | P | PrD | N | 0.34 | N | N | -8.43 |
| CM056063 | W267R | D | C65 | NN | P | PrD | N | 0.23 | N | N | -7,66 |
| CM045553 | F268S | D | C65 | NN | P | PrD | D | 0.19 | N | N | -5.43 |
| CM100897 | F268L | N | C15 | N | UN | B | D | 0.29 | D | D | -4.33 |
| VAR_033095 | F268C | D | C65 | NN | P | PrD | D | 0.56 | D | D | -4.46 |
| CM112117 | A269P | N | C25 | N | Un | PrD | N | 0.67 | D | D | -4.35 |
| VAR_010562/CM981901 | R271W | D | C65 | NN | P | PrD | D | 0.54 | D | D | -5.73 |
| VAR_010563/CM991168 | R272C | D | C65 | NN | P | PrD | D | 0.67 | D | D | -5.72 |
| VAR_003759/rs137853238/CM971459 | R272H | D | C25 | NN | P | PrD | D | 0.29 | D | D | -3.9 |
| CM082859 | R272S | D | C65 | NN | UN | PrD | N | 0 | N | D | -3.45 |
| VAR_033096 | K273E | D | C55 | NN | P | PrD | D | 0.59 | D | D | -3.78 |
| CM064298 | K273N | D | C65 | NN | UN | PrD | N | 0.02 | N | N | -5.44 |
| rs199890776 | E275A | N | C65 | NN | P | PrD | D | 0.85 | D | N | -3.73 |
| CM082855 | A276G | D | C55 | NN | N | B | N | 0.76 | N | D | -3.65 |
| rs137853245/CM030527 | A276D | D | C65 | N | P | PrD | D | 0.53 | D | D | -4.27 |
| CM082845 | L281P | D | C65 | N | P | PoD | N | 0.65 | N | D | -7.45 |
| rs267603343 | P289L | N | C65 | N | UN | PoD | D | 0.13 | N | N | -4.24 |
| rs193922606/CM002866 | P291R | N | C65 | NN | UN | B | D | 1. 00 | UC | N | -4.11 |
| CM082826 | P291T | N | C35 | N | P | B | N | 0.05 | D | N | -3.56 |
| rs151256267/CM082856 | P291S | N | C65 | N | UN | B | N | 1. 00 | UC | N | -4.27 |
| CM995176 | A301T | D | C55 | NN | P | PrD | D | 0 | D | N | -1.16 |
| CM082835 | P308L | N | C65 | NN | UN | PoD | N | 0.5 | D | D | -2.43 |
| CM050752 | A311D | N | C65 | NN | UN | PoD | N | 0.35 | D | D | -3.44 |
| rs267603344 | P314L | N | C65 | NN | UN | PoD | D | 0.14 | N | N | -4.55 |
| VAR_010564/rs137853240/CM993622 | G319S | N | C55 | NN | UN | PoD | D | 1. 00 | D | N | -5.41 |
| rs140491072/CM082873 | Y322C | N | C65 | NN | UN | PrD | D | 0.18 | D | D | -5.79 |
| CM082810 | S328R | N | C65 | NN | UN | PrD | N | 0.36 | D | N | -3.45 |
| CM121653 | L348P | D | C65 | N | P | PrD | N | 0.29 | N | D | -6.44 |
| CM002867 | T354M | N | C65 | NN | P | PrD | D | 0.45 | D | D | -2.34 |
| CM050753 | P379R | N | C65 | N | P | B | D | 0.05 | D | N | -1.18 |
| CM082860 | A373G | D | C55 | N | N | PoD | N | 0.82 | N | N | -5.45 |
| rs371717826/CM056993 | P379H | N | C65 | NN | P | PrD | D | 0.07 | D | D | -5.19 |
| CM082831 | P379S | N | C65 | NN | UN | B | D | 0.29 | N | N | -4.56 |
| CM082829 | P379T | N | C35 | N | P | PoD | N | 0.56 | D | D | -3.46 |
| rs111496840 | V380D | D | C65 | N | P | PrD | D | 0.62 | D | D | -4.72 |
| rs115080759/CM082848 | L389V | N | C25 | NN | UN | PoD | D | 0.35 | N | N | -3.45 |
| rs376044120/CM082834 | P409H | N | C65 | NN | P | PrD | D | 0.07 | D | N | -4.56 |
| CM064304 | M412T | D | C65 | NN | UN | PrD | D | 0.56 | N | D | -3.44 |
| VAR_010565/CM993760 | G415R | N | C65 | N | P | PrD | D | 0.51 | D | N | -5.57 |
| rs377243343 | G417C | N | C25 | N | UN | PrD | D | 0.18 | D | N | -4.47 |
| rs193922577 | L422P | N | C65 | NN | P | B | D | 0.43 | D | N | -5.55 |
| CM082836 | P424L | N | C65 | NN | UN | PoD | D | 0.29 | N | N | -3.45 |
| VAR_012485/CM993817 | S432C | N | C65 | NN | UN | PrD | D | 0.18 | D | N | -5.59 |
| CM002868 | T433I | N | C65 | NN | P | PoD | N | 0.29 | D | N | -5.44 |
| rs371544082 | T441K | N | C65 | NN | UN | B | D | 0.46 | N | N | -4.36 |
| VAR_003760/rs137853236/CM064310 | P447L | N | C65 | NN | P | PrD | D | 0.16 | D | D | -5.77 |
| CM064301 | T457I | N | C65 | NN | P | PoD | D | 0.45 | D | N | -3.44 |
| rs78886627/CM082875 | S465P | N | C65 | NN | UN | B | D | 0.44 | N | N | -4.5 |
| CM082833 | P476L |  | C35 | N | UN | PrD | N | 0.43 | N | N | -3.44 |
| rs201811844 | H469Y | N | C65 | NN | P | PrD | D | 0.03 | D | N | -4.67 |
| rs193922580/CM092844 | P475L | N | C65 | NN | UN | PrD | D | 0.2 | D | N | -4.45 |
| VAR_007906/rs2464196/CM067474 | S487N | N | C45 | N | UN | B | D | 0.4 | N | N | -4.39 |
| CM082847 | F489V | D | C45 | N | P | B | D | N | N | N | -3.24 |
| CM128623 | M490R | D | C65 | NN | P | PrD | D | D | N | D | -5.67 |
| CM995174 | T492I | N | C65 | NN | UN | PoD | D | 0.02 | N | N | -3.44 |
| CM995175 | S498R | N | C65 | N | P | PrD | D | 0.06 | D | D | -5.46 |
| CM082827 | P499S | N | C65 | N | UN | PoD | N | 0.54 | N | N | -5.97 |
| CM082822 | H500N | N | C65 | NN | P | B | N | 0.44 | N | N | -6.33 |
| rs371807951/CM035682 | A501T | N | C55 | NN | UN | PrD | D | 0.65 | N | N | -5.54 |
| CM082823 | H505N | N | C65 | N | P | PoD | D | 0.4 | N | N | -6.78 |
| CM082841 | G508K | D | C65 | NN | P | B | N | 0.3 | N | N | -4.22 |
| rs143592417 | Q511R | N | C35 | NN | UN | PrD | D | 0.55 | N | N | -4.52 |
| VAR_010566/rs202039659/CM013295 | H514R | N | C25 | NN | P | PoD | D | 0.55 | N | N | -4.41 |
| CM082807 | T515K | D | C55 | N | N | PoD | D | 0.54 | D | N | -4.35 |
| rs200639058 | P519S | N | C0 | NN | UN | PrD | D | 0.29 | N | N | -4.18 |
| VAR_010567/CM971460 | P519L | N | C0 | NN | UN | PrD | D | 0.29 | N | N | -4.51 |
| CM112123 | T521A | D | C65 | NN | UN | PoD | D | 0.02 | N | D | -6.53 |
| CM023590 | T521I | D | C45 | NN | N | PoD | N | 0.44 | N | N | -3.74 |
| CM030528 | M522V | N | C15 | NN | UN | B | N | 0.67 | N | N | -4.44 |
| rs137853246 | S531T | N | C55 | N | UN | B | D | 0.82 | N | N | -4.38 |
| rs372624970 | T537M | N | C65 | NN | UN | PrD | D | 0.43 | D | N | -4.58 |
| VAR_010568 | T537R | N | C65 | NN | P | PrD | D | 0.43 | D | N | -4.55 |
| rs193922587 | L555F | N | C15 | NN | UN | B | D | 0.71 | N | N | -3.03 |
| rs144674840 | P568L | N | C65 | N | P | B | D | 0. 31 | UC | N | -2.74 |
| VAR_010569 | G574S | N | C55 | NN | N | B | D | 0.96 | UC | N | -2.54 |
| rs376832928 | H577D | N | C65 | NN | UN | PoD | D | 0.52 | UC | N | -3.77 |
| rs193922589 | H582R | N | C25 | NN | UN | PrD | D | 0.41 | UC | N | -2.87 |
| VAR_003761/rs137853242 | R583G | N | C65 | NN | UN | PrD | D | 0.61 | UC | N | -3.46 |
| VAR_010570/rs137853242 | R583Q | N | C35 | NN | UN | PrD | D | 0.61 | UC | N | -3.44 |
| rs373857078 | A586T | N | C0 | N | N | B | N | 0.68 | UC | N | -2.63 |
| rs200120574 | S593T | N | C55 | NN | UN | B | D | 0.6 | UC | N | -2.98 |
| VAR_010571 | S594I | N | C65 | NN | P | B | D | 0.23 | UC | N | -3.24 |
| rs375702866 | S616R | N | C65 | NN | UN | B | D | 0.35 | UC | N | -2.76 |
| rs146855738 | V617I | N | C25 | NN | UN | B | N | 0.2 | UC | N | -2.86 |
| VAR_012486/rs193922591/CM993818 | I618M | N | C0 | NN | UN | B | D | 0.11 | UC | N | -3.58 |
| VAR_010572/CM981903 | E619K | N | C55 | NN | P | PrD | D | 0.76 | UC | N | -2.99 |
| VAR_010573/rs137853241/CM971462 | T620I | N | C65 | NN | UN | B | D | 0.19 | UC | N | -2.95 |

**^N- Neutral, UN- Unknown, C-Class, NN- Non-Neutral, P-Pathogenic, B-Benign, D- Deleterious, PrD- Probably Damaging, PoD- Possibly Damaging, UC- Unclassified^**
