# Supplementary material for: Determining the role of missense mutations in the POU domain of HNF1A that reduce the DNA-binding affinity: A computational approach
Source: PLoS One. 2017 Apr 14;12(4):e0174953. doi: 10.1371/journal.pone.0174953 (PMC5391926; doi:10.1371/journal.pone.0174953)
Supplement: S2 Table — (DOCX) [file pone.0174953.s009.docx]

**S2 Table.** Comparative analysis of the DNA-binding sites of HNF1A missense mutations predicted by PDBsum and BindN+

| **Mutation** | **PDBSum** | **BindN+** |
| --- | --- | --- |
| M1V | Negative (-) | Negative (-) |
| L5P | Negative (-) | Negative (-) |
| L12H | Negative (-) | Negative (-) |
| G20R | Negative (-) | Negative (-) |
| I27L | Negative (-) | Negative (-) |
| G31D | Negative (-) | Negative (-) |
| G34R | Negative (-) | Negative (-) |
| G47R | Negative (-) | Negative (-) |
| E48K | Negative (-) | Negative (-) |
| G52A | Negative (-) | Negative (-) |
| N62S | Negative (-) | Negative (-) |
| R68Q | Negative (-) | Positive (+) |
| E79V | Negative (-) | Negative (-) |
| D80Y | Negative (-) | Negative (-) |
| L86H | Negative (-) | Negative (-) |
| P94L | Negative (-) | Negative (-) |
| A97V | Negative (-) | Negative (-) |
| A98V | Negative (-) | Negative (-) |
| V103M | Negative (-) | Negative (-) |
| L107R | Negative (-) | Negative (-) |
| P112L | Negative (-) | Negative (-) |
| R114H | Negative (-) | Negative (-) |
| K117E | Negative (-) | Positive (+) |
| Y122C | Negative (-) | Positive (+) |
| N127Y | Negative (-) | Negative (-) |
| I128N | Negative (-) | Negative (-) |
| P129T | Positive (+) | Negative (-) |
| R131Q | Positive (+) | Positive (+) |
| R131W | Positive (+) | Positive (+) |
| V133M | Negative (-) | Negative (-) |
| S142F | Positive (+) | Positive (+) |
| H143Y | Positive (+) | Positive (+) |
| H147Q | Negative (-) | Negative (-) |
| K155R | Positive (+) | Positive (+) |
| T156M | Negative (-) | Positive (+) |
| K158N | Positive (+) | Positive (+) |
| R159Q | Negative (-) | Positive (+) |
| R159W | Negative (-) | Positive (+) |
| A161T | Negative (-) | Negative (-) |
| W165C | Negative (-) | Positive (+) |
| V167I | Negative (-) | Negative (-) |
| R168H | Negative (-) | Positive (+) |
| A174V | Negative (-) | Negative (-) |
| Q175R | Negative (-) | Negative (-) |
| G191D | Negative (-) | Negative (-) |
| T196A | Negative (-) | Negative (-) |
| R200W | Negative (-) | Positive (+) |
| R203C | Positive (+) | Positive (+) |
| R203H | Positive (+) | Positive (+) |
| K205Q | Positive (+) | Positive (+) |
| W206C | Negative (-) | Positive (+) |
| W206L | Negative (-) | Positive (+) |
| P224S | Positive (+) | Negative (-) |
| R229Q | Negative (-) | Positive (+) |
| N237S | Negative (-) | Negative (-) |
| C241G | Negative (-) | Negative (-) |
| R244I | Negative (-) | Negative (-) |
| R244G | Negative (-) | Positive (+) |
| G245V | Negative (-) | Negative (-) |
| Q250P | Negative (-) | Positive (+) |
| L254M | Negative (-) | Negative (-) |
| V259D | Negative (-) | Negative (-) |
| T260M | Negative (-) | Negative (-) |
| R263C | Positive (+) | Positive (+) |
| V264F | Negative (-) | Negative (-) |
| F268S | Negative (-) | Negative (-) |
| F268C | Negative (-) | Negative (-) |
| R271W | Negative (-) | Positive (+) |
| R272C | Positive (+) | Positive (+) |
| R272H | Positive (+) | Positive (+) |
| K273E | Positive (+) | Positive (+) |
| E275A | Negative (-) | Negative (-) |
| A276D | Negative (-) | Negative (-) |
| P289L | Negative (-) | Negative (-) |
| P291R | Negative (-) | Negative (-) |
| P291S | Negative (-) | Negative (-) |
| P314L | Negative (-) | Negative (-) |
| G319S | Negative (-) | Negative (-) |
| Y322C | Negative (-) | Positive (+) |
| P379H | Negative (-) | Negative (-) |
| V380D | Negative (-) | Negative (-) |
| L389V | Negative (-) | Negative (-) |
| P409H | Negative (-) | Negative (-) |
| G415R | Negative (-) | Negative (-) |
| G417C | Negative (-) | Negative (-) |
| L422P | Negative (-) | Negative (-) |
| S432C | Negative (-) | Positive (+) |
| T441K | Negative (-) | Positive (+) |
| P447L | Negative (-) | Negative (-) |
| S465P | Negative (-) | Positive (+) |
| H469Y | Negative (-) | Negative (-) |
| P475L | Negative (-) | Negative (-) |
| S487N | Negative (-) | Positive (+) |
| A501T | Negative (-) | Negative (-) |
| Q511R | Negative (-) | Negative (-) |
| H514R | Negative (-) | Negative (-) |
| P519S | Negative (-) | Negative (-) |
| P519L | Negative (-) | Negative (-) |
| S531T | Negative (-) | Positive (+) |
| T537M | Negative (-) | Positive (+) |
| T537R | Negative (-) | Positive (+) |
| L555F | Negative (-) | Negative (-) |
| P568L | Negative (-) | Negative (-) |
| G574S | Negative (-) | Negative (-) |
| H577D | Negative (-) | Negative (-) |
| H582R | Negative (-) | Negative (-) |
| R583G | Negative (-) | Positive (+) |
| R583Q | Negative (-) | Positive (+) |
| A586T | Negative (-) | Negative (-) |
| S593T | Negative (-) | Positive (+) |
| S594I | Negative (-) | Positive (+) |
| S616R | Negative (-) | Negative (-) |
| V617I | Negative (-) | Negative (-) |
| I618M | Negative (-) | Negative (-) |
| E619K | Negative (-) | Negative (-) |
| T620I | Negative (-) | Negative (-) |
