# Supplementary material for: Determining the role of missense mutations in the POU domain of HNF1A that reduce the DNA-binding affinity: A computational approach
Source: PLoS One. 2017 Apr 14;12(4):e0174953. doi: 10.1371/journal.pone.0174953 (PMC5391926; doi:10.1371/journal.pone.0174953)
Supplement: S3 Table — (DOCX) [file pone.0174953.s010.docx]

**S3 Table.** Interactions observed between the DNA and the protein in native and R131W, R131Q, and R203C mutant complexes

| **Interactions** | **Native** | **R131W** | **R131Q** | **R203C** |
| --- | --- | --- | --- | --- |
| Number of interacting residues molecule 1 (protein) | 69 | 69 | 72 | 72 |
| Number of interacting residues molecule 2 (DNA) | 33 | 33 | 34 | 34 |
| Number of hydrophobic- hydrophilic interaction | 50 | 50 | 54 | 54 |
| Number of hydrophilic-hydrophilic interaction | 181 | 183 | 201 | 202 |
| Number of hydrophobic- hydrophobic interaction | 0 | 0 | 0 | 0 |
