# Supplementary material for: Determining the role of missense mutations in the POU domain of HNF1A that reduce the DNA-binding affinity: A computational approach
Source: PLoS One. 2017 Apr 14;12(4):e0174953. doi: 10.1371/journal.pone.0174953 (PMC5391926; doi:10.1371/journal.pone.0174953)
Supplement: S4 Table — (DOCX) [file pone.0174953.s011.docx]

**S4 Table.** Number of salt bridges formation in the native and mutant (R131W, R131Q, and R203C) complexes.

| **Protein Complexes** | **Number of Salt Bridges** | **Residue 1** | **Residue 2** | **Distance (Å)** |
| --- | --- | --- | --- | --- |
| Native | 13 | NE2 HIS126  NZ LYS169  NH1 ARG238  NH2 ARG238  NH2 ARG244  ND1 HIS126  NH2 ARG131  NZ LYS169  NZ LYS222  NZ LYS226  NZ LYS226  NH2 ARG 244  NH1 ARG271 | OE1 GLU172  OD1 GLU132  OE1 GLU234  OE1 GLU 234  OE1 GLU 240  OE1 GLU 172  OD2 ASP 135  OE1 GLU 132  OE1 GLU275  OE1 GLU230  OE1 GLU261  OE1 GLU 240  OE1 GLU 275 | 3.71  2.58  3.52  3.55  2.32  3.78  3.20  2.9  3.75  3.52  3.66  2.28  2.07 |
| R131W | 13 | NE2 HIS 126  NZ LYS 169  NH1 ARG 238  NH2 ARG 238  NH2 ARG 244  ND1 HIS 126  NH2 ARG 131  NZ LYS 169  NZ LYS 222  NZ LYS 226  NZ LYZ 226  NH2 ARG 244  NH1 ARG 271 | OE1 GLU 172  OE1 GLU 132  OE1 GLU 234  OE1 GLU 234  OE1 GLU 240  OE1 GLU 172  OE1 ASP 135  OE1 GLU 132  OE1 GLU 275  OE1 GLU 230  OE1 GLU 261  OE1 GLU 240  OE1 GLU 275 | 3.14  2.96  3.82  3.91  3.09  3.74  3.43  3.02  3.63  3.56  3.40  3.42  3.18 |
| R131Q | 13 | NE2 HIS 126  NZ LYS 169  NH1 ARG 238  NH2 ARG 238  NH2 ARG 244  ND1 HIS 126  NH2 ARG 131  NZ LYS 169  NZ LYS 222  NZ LYS 226  NZ LYZ 226  NH2 ARG 244  NH1 ARG 271 | OE1 GLU 172  OE1 GLU 132  OE1 GLU 234  OE1 GLU 234  OE1 GLU 240  OE1 GLU 172  OE1 ASP 135  OE1 GLU 132  OE1 GLU 275  OE1 GLU 230  OE1 GLU 261  OE1 GLU 240  OE1 GLU 275 | 3.71  2.58  3.52  3.55  2.32  3.78  3.20  2.99  3.75  3.52  3.66  2.28  3.07 |
| R203C | 5 | NE2 HIS 126  NZ LYS 169  NH1 ARG 238  NH2 ARG 238  NH2 ARG 244 | OE1 GLU 172  OE1 GLU 132  OE1 GLU 234  OE1 GLU234  OE1 GLU 240 | 3.71  2.58  3.52  3.55  2.32 |
